# Snapshots of the Stopped Polymerization of a Hindered Isocyanide within the Coordination Sphere of Ni(II)

**DOI:** 10.1021/acs.inorgchem.4c05461

**Published:** 2025-03-07

**Authors:** Clara Del Carmen-Rodríguez, Lucía Álvarez-Miguel, Celedonio M. Álvarez, Raúl García-Rodríguez, Daniel Miguel

**Affiliations:** a GIR MIOMET/IU CINQUIMA/Química Inorgánica, Facultad de Ciencias Universidad de Valladolid, Valladolid E-47011, Spain; b SOSCATCOM Group. Departamento de Química Orgánica y Química Inorgánica, Facultad de Farmacia and Instituto de Investigación Química “Andrés M. del Río” (IQAR) Universidad de Alcalá Campus Universitario, Alcalá de Henares, Madrid 28871, Spain

## Abstract

The use of cationic Ni­(II) complexes containing dialkyldithiophosphate ligands in conjunction with sterically hindered isocyanides suppresses polymerization, allowing the formation of well-defined monomeric cationic Ni­(II) complexes **3** that result from the coupling of three isocyanides. These complexes have been characterized, including X-ray structure determination, and represent a snapshot of the first steps of the polymerization of isocyanide. Studies via X-ray, IR, and NMR seem to indicate that the key active species in the Ni­(II)-catalyzed isocyanide polymerization and its so-called “merry-go-round” mechanism is not a carbene, as has been proposed, but actually a formamidinyl species. The use of the most sterically congested set of ligands enabled the isolation of the intermediate species **4c**, which contains only two coupled isocyanides and can be used in the stepwise and controlled synthesis of a rare mixed Ni­(II) complex **5** by using two different isocyanides.

## Graphical abstract

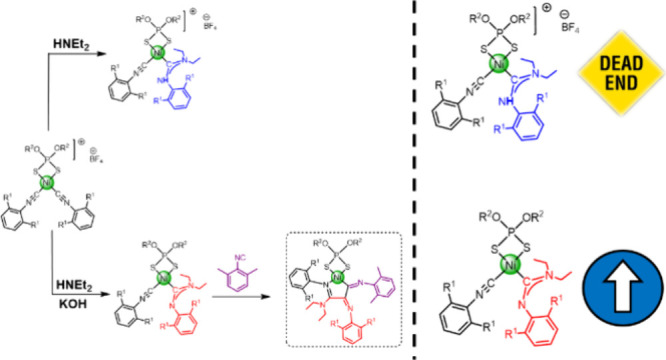

## Introduction

The Ni­(II)-catalyzed polymerization of isocyanides is an important reaction to provide chiral helical polyisocyanide (i.e., polyiminomethylene) polymers^1–9^ via rapid living polymerization processes,^3,10,11^ which can later undergo block copolymerization^7,12–15^ and graft polymerization.^16,17^ This reactivity was first demonstrated in the pioneering work of Grundman^18^ and Otsuka et al.,^19^ and later extensively studied by Drenth and Nolte^9,20–22^ and Deeming and Novak.^23^

However, the catalytic activity of Ni­(II) toward isocyanides represents an obstacle to the preparation of “well-defined” Ni­(II) isocyanide complexes, as the formation of (in this case) undesired polymeric materials often occurs. Thus, Ni­(II) isocyanide complexes remain relatively scarce compared to their heavier Pd and Pt congeners^24–26^ and there have been only a few studies of the reactivity of isocyanide coordinated to Ni­(II),^27–30^ apart from those dealing with the Ni­(II)-catalyzed isocyanide polymerization process and its so-called “merry-go-round” mechanism (*see* Scheme 1).^22,31,32^ This mechanism is initiated by the attack of a primary or secondary amine on a coordinated isocyanide in a cationic square-planar tetrakis-isocyanide Ni­(II) complex to produce a carbene ligand. This reaction is well established and has been extensively used to obtain acyclic diamino carbene ligands.^24,25^ The resulting nickel carbene has been proposed to be the key active intermediate in the process; it is thought that this nickel carbene goes on to attack an adjacent coordinated isocyanide, triggering polymerization via successive attacks around the coordination sphere of Ni. However, the instability of Ni­(II) isocyanides and their marked tendency to polymerize have complicated detailed mechanistic studies.^22,31,32^

**Scheme 1:**
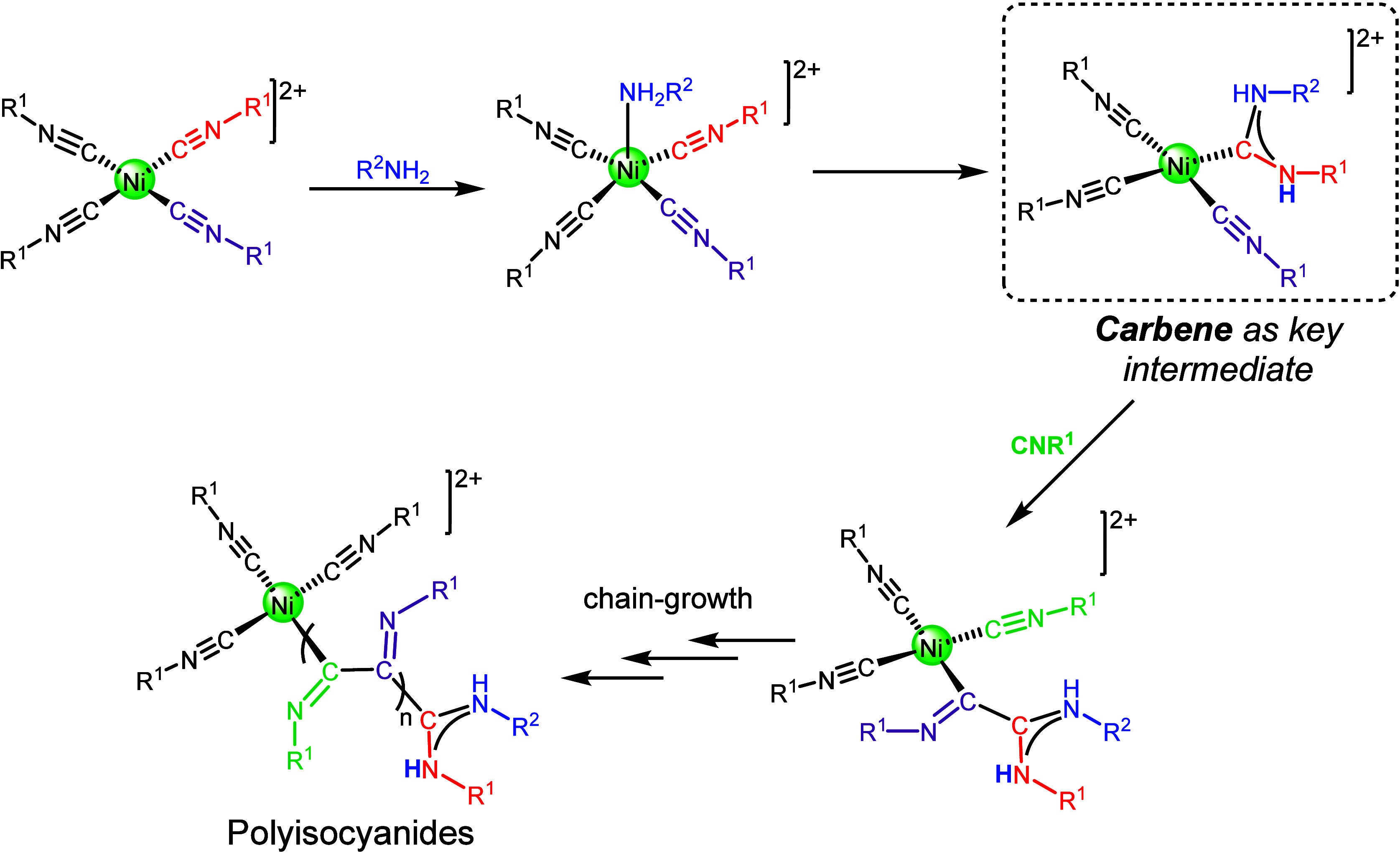
Merry-Go-Round Mechanism to Obtain Polyisocyanides in Which the Carbene Is Proposed to Be the Key Active Intermediate^a^

a Adapted from ref 31 with permission. Copyright 2007 John Wiley and Sons.

We have recently developed a convenient method to access scarce Ni­(II)-acyclic diaminocarbenes (ADCs) through the well-known approach of nucleophilic addition of a secondary amine to a coordinated isocyanide.^33^ While Pd­(II) and Pt­(II) carbenes have been widely obtained using this method, access to their Ni­(II) counterparts had been dramatically restricted due to the poor stability of suitable Ni­(II) complexes as starting materials. We found that bis-isocyanide Ni­(II) complexes featuring mono- or dialkyl-dithiophosphate as ancillary ligands were ideal precursors for the preparation of both neutral and cationic Ni-ADCs through reaction with secondary amines (Scheme 2). The presence of dithiophosphate and soft isocyanide ligands was key to stabilizing the square-planar Ni­(II) carbenes, resulting in air-stable Ni-ADC complexes. However, while neutral Ni-ADCs were isolated in very good yields (typically 90%, Scheme 2 *a*), the preparation of cationic Ni-ADCs (Scheme 2 *b*) was complicated by the formation of unidentified red side products, resulting in poor yields of the corresponding carbenes.^33^

**Scheme 2:**
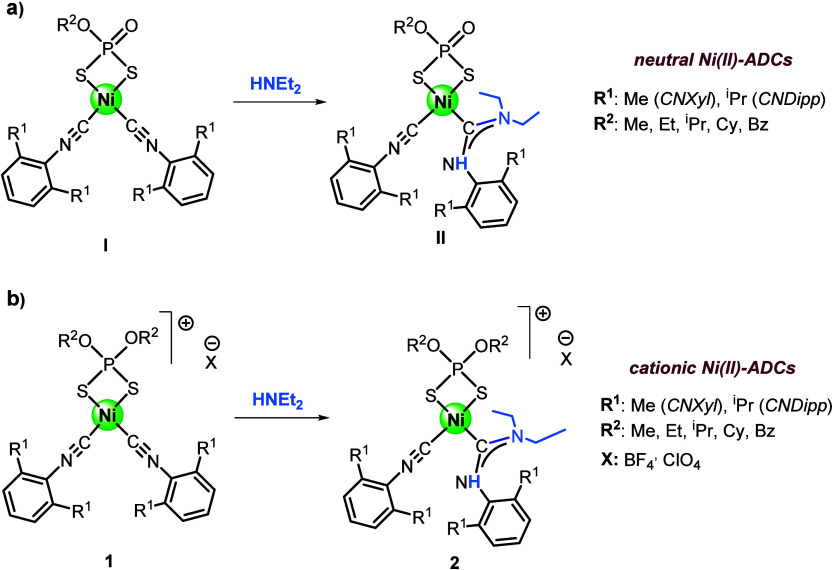
Synthetic Strategy to Obtain Neutral (a) and Cationic (b) Scarce Ni­(II) Acyclic Carbenes (Ni-ADCs) with Dithiophosphate Ligands Recently Developed in Our Group^33^

In the present work, we have identified these elusive byproducts as monomeric cationic Ni­(II) complexes that result from the coupling of three isocyanides. Isolation and characterization of these complexes was accomplished by using bulky isocyanides and dithiophosphate ligands, thus providing steric shielding at the Ni center and preventing further extended polymerization from proceeding. The isolation and crystallographic characterization of these complexes provides snapshots of the initial steps of the important isocyanide polymerization. This opens the way for the control of the C–C coupling in a step-by-step fashion, which allowed the preparation of a mixed-isocyanide compound in a stepwise manner. With this information in hand, we have examined the mechanism of the formation of these complexes via isocyanide coupling in detail. Surprisingly, our results seem to contradict the prevailing assumption that the nickel carbene is the active species in the polymerization process and shed new light on the nature of the active species in the so-called merry-go-round mechanism, which could have important implications for the synthesis of polyisocyanide materials.

## Results and Discussion

As noted in the introduction, we have recently found that cationic bis-isocyanide complexes **1** featuring dialkyldithiophosphate ancillary ligands can be used as precursors to obtain cationic acyclic diaminocarbene complexes (ADCs) **2** by reaction with diethylamine (*see* Scheme 2 *b*). These monocarbenes, which contain a dithiophosphate ligand and one remaining isocyanide, were isolated as yellow solids. However, in contrast to neutral ADCs **II** (Scheme 2 *a*), cationic ADC_S_ **2** could only be isolated in low-to-moderate yields (ca. 50%), leaving behind intense deep-red-colored mother liquors, evidencing the formation of additional side products. We suspected that the higher electrophilicity of the isocyanide carbon in cationic ADCs as compared to the neutral ACDs (as evidenced from the higher ν­(CN) in the IR spectrum)^33^ could make them more prone to nucleophilic attack, leading to the formation of this red species. To explore this intriguing observation further, the reaction of **1a** and 2 equiv of NHEt_2_ in CH_2_Cl_2_ (30 min at room temperature) resulted in the formation of cationic carbene **2a**, in which one molecule of amine was added across the CN bond to form a carbene ligand, which could be isolated as yellow microcrystals by precipitation with diethyl ether. The deep red mother liquor was separated, further concentrated in MeOH and cooled at −25 °C for 24 h. To our delight, we were able to obtain red crystals of neutral complex **3a** suitable for X-ray crystallography in ca. 10% crystalline yield (*see* Scheme 3 and later for the optimized conditions for the synthesis of **3a**).

**Scheme 3:**
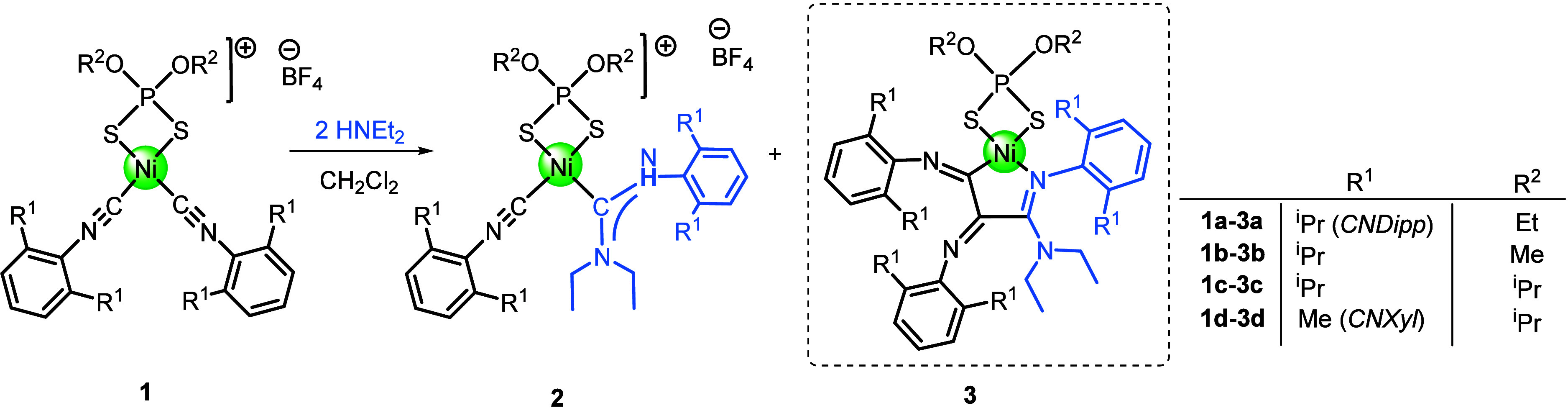
Reaction of Cationic Bis-isocyanide Complexes **1** to Give Cationic Carbene Complexes **2** and C–C Coupled Neutral Complexes **3** ^a^

a The C,N chelating ligand in **3** includes two new C–C bonds involving the carbon atoms of the original isocyanides.

The single-crystal X-ray structure reveals that **3a** arises from the coupling of three isocyanides, leading to the formation of a chelating *k* ^2^(C, N) ligand that, together with the Ni atom, forms a five-membered chelate ring or nickelmetallacycle (5-NiCy). The formation of this metallacycle involves the formation of two new C–C bonds and incorporates the NEt_2_ group in the structure. The nitrogens of two of the iminoacyl groups are not included in the metallacycle, while for the third iminoacyl group, both the nitrogen and the carbon (which is attached to the NEt_2_ group) are incorporated into the 5-NiCy. The diethyldithiophosphate ligand bonded *k* ^2^(S,S′) completes the square-planar environment around the Ni­(II) center (Figure 1). The ^1^H NMR spectrum in CDCl_3_ shows the expected resonances corresponding to the three inserted isocyanide groups, along with the ethyl groups in the dithiophosphate ligand (δ 3.81 and 1.12 ppm) and the NEt_2_ moiety attached to the nickelmetallacycle (δ 3.41 and 1.06 ppm). The ^13^C NMR spectrum is more informative, showing three quaternary carbon resonances at 182.3, 160.9, and 158.5 ppm corresponding to the metallacyclic skeleton. Finally, the ^31^P­{^1^H} NMR signal of **3a** (δ 97.1 ppm) appears significantly downfield with respect to that of **1a** (δ 89.7 ppm) and the ADC carbene **2a** (δ 92.0 ppm).^33^

**Figure 1:**
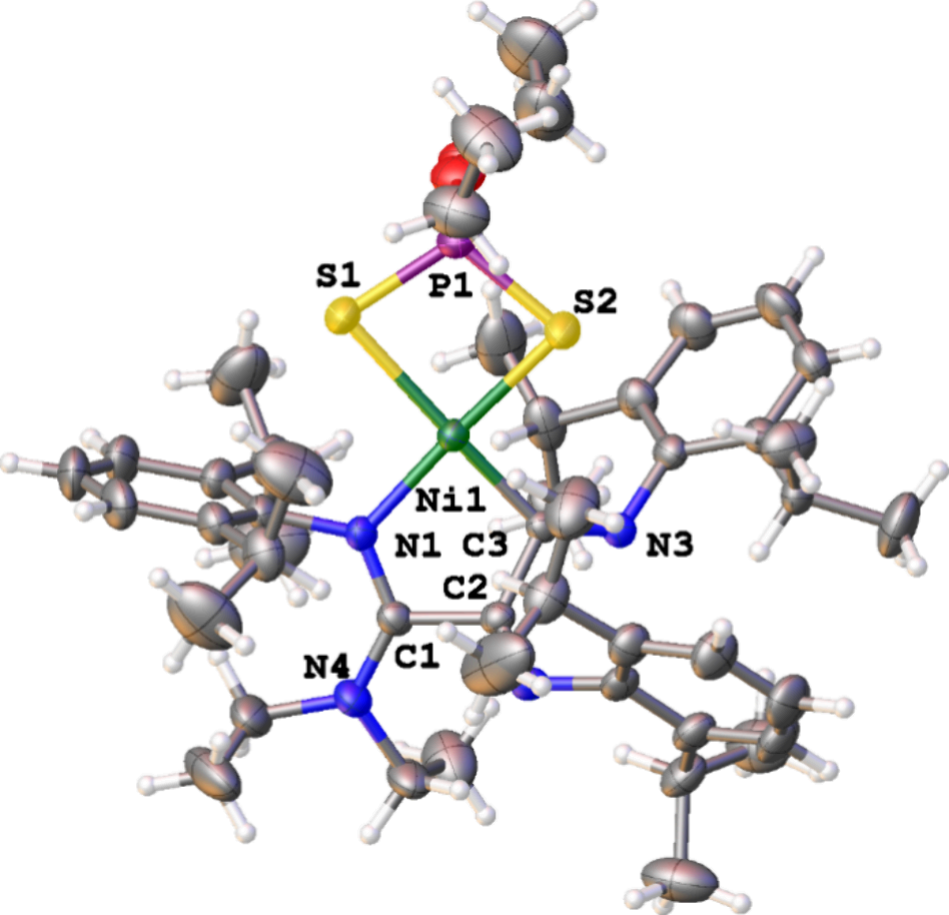
X-ray diffraction structure of complex **3a**. Selected bond lengths (Å) and angles (deg): Ni(1)–S(1): 2.3438(9), Ni(1)–S(2): 2.2191(9), Ni(1)–N(1): 1.944(2), Ni(1)–C(3): 1.881(3), S(1)–Ni(1)–S(2): 87.34(3), N(1)–Ni(1)–C(3): 85.41(10).

Similar results were observed starting from **1b**–**d**, which feature different dialkyldithiophosphate ligands (R^2^ = Me, ^i^Pr) and 2,6-disubstituted aryl isocyanides (R^1^ = Me, ^i^Pr). In all cases, reaction with 2 equiv of NHEt_2_ (30 min at room temperature in CH_2_Cl_2_) afforded the expected cationic Ni-ADCs **2b**–**d** along with a small fraction of red complexes corresponding to neutral **3b**–**d** that could be isolated and fully characterized including structural confirmation by X-ray crystallography (*see* Scheme 3 and X-ray Crystallographic Studies in the *SI*). Complexes **3** contain a five-membered metallacycle ring that arises from the coupling of the three 2,6-disubstituted aryl isocyanides. Since the isocyanide source **1** contains only two isocyanides per Ni atom, the third unit must be scavenged from an additional complex **1** or another intermediate species, thus lowering the final yield, although the exact details of this process are unclear at this stage (see later for the optimized conditions to obtain complexes **3**, which require the use of an additional equivalent of isocyanide, Scheme 5).

Importantly, complexes **3** can be viewed as a snapshot of the first steps of the polymerization for the well-known and important polymerization of isocyanides to give poly­(iminomethylene). The formation of **3** includes the coupling of isocyanides, which is the route proposed for this polymerization, but which has been suppressed in the formation of **3**. The presence of bulky substituents in the group attached to the isocyanide N atom and the dialkyldithiophosphate ligands appears to impede the polymerization. In the case of complexes **3**, the bulkiness of the groups at the 2,6-positions of the phenyl ring (R^1^ = ^i^Pr or Me) is not sufficient to completely block C–C coupling, but produces sufficient steric hindrance to prevent the extended polymerization, allowing the isolation of complexes **3**. In order to further prove this idea, we reacted 2 mmol of the bulky isocyanide 2,6-dimethyl-phenyl isocyanide (CNXyl), which features methyl groups at the ortho positions, with complex **1d** (10 mol %, 0.2 mmol), NHEt_2_ (20 mol %, 0.4 mmol) and KOH in excess (10 mol %, 0.2 mmol) in 15 mL of DCM (KOH was added to ensure the formation of the formamidinyl species **C–F**, *vide infra*). IR monitoring of the reaction showed no changes in the CN band after 26 h at room temperature. Under the same conditions, the use of sterically unhindered 4-phenylazophenylisocyanide, in which the substituent is located at the remote para position and the ortho position is unsubstituted, showed fast polymerization, as evidenced by the disappearance of the CN band and the formation of a viscous mixture within minutes. Remarkably, even the use of only 1.5 mol % of complex **1d** provided the same results.

Having observed the formation of carbene **2** along with complexes **3**, we sought to study the role of the carbene ligand in the promotion of the C–C coupling. This is an important point since an intermediate carbene has been proposed in the so-called “merry-go-round” mechanism (*see* Scheme 1).^22,31,32^ We first tested whether the isolated carbene alone would evolve to give the final product. In an independent experiment, isolated carbene **2c** was subjected to forcing conditions (including reflux in CHCl_3_ for 24 h or microwave heating at 140 °C, 2 h, *see* SI, Figure S1 and S2) but no C–C coupling was observed. Since the formation of complexes **3** occurs at room temperature, this implies that the carbene complexes **2** are truly final side-products rather than active direct intermediates in the coupling process as has been generally asserted.

A schematic view of the pathway leading from **1** to **3** is presented in Scheme 4, based on the experimental data discussed below. The process is initiated by the nucleophilic attack of the amine on the electrophilic isocyanide carbon activated by coordination to Ni. The first product of the addition (**A**) loses the proton at the N atom to give a C-bonded formamidinyl ligand (**C–F**), which can be considered a deprotonated carbene. This is favored by the presence of an excess of amine, which appears to play a role as a proton abstractor of **A** in this step. In fact, nucleophilic attack of amines on coordinated isocyanides followed by proton migration is a well-known route to produce carbenes, and studies on Pd­(II) and Pt­(II) systems, including recent and detailed DFT calculations, have shown that this proton transfer occurs in a stepwise fashion via deprotonation and protonation steps assisted by a second molecule of amine.^34^ Moreover, *in situ* NMR studies show that the reaction of **1** with just one equivalent of NHEt_2_ leads to a sluggish and incomplete transformation of **1** into the corresponding carbene **2** as the only species observed by NMR. It is only in the presence of two equivalents of amine that a full (and fast) conversion of **1** into **2** is observed, albeit accompanied by the formation of small amounts of **3** (*see* SI, Figure S3).

**Scheme 4:**
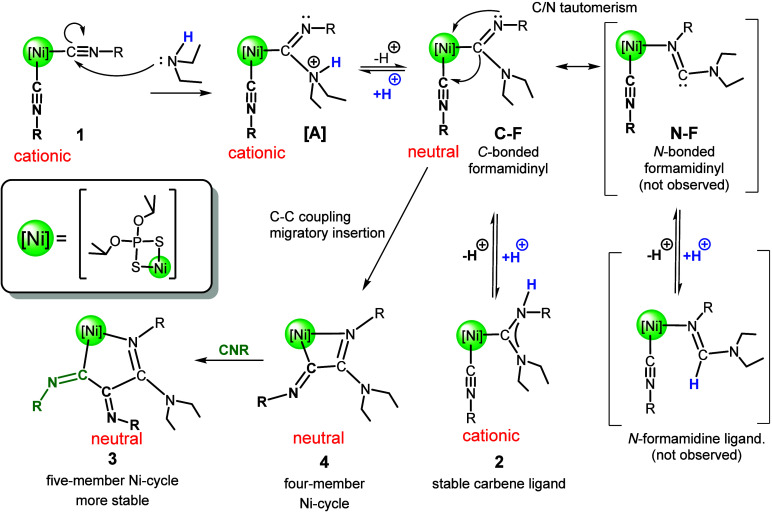
Proposed Reaction Path Leading from Isocyanide Complexes **1** to Carbene **2** and Five-Member Metallacycle **3** via the Intermediate **4** with a Four-Membered Metallacycle

Previous examples of *N*-formamidine complexes resulting from the deprotonation of a coordinated diaminocarbene have been reported and characterized.^35,36^ In some cases, the deprotonation is accompanied by a shift from *C*- to *N*-coordination. In the present study, the presence of the N-bonded tautomer **N–F** cannot be completely ruled out, but this is not relevant since the C-bonded carbene complexes **2** are the only species produced upon protonation, as we will demonstrate below. Our results suggest that formamidinyl species **C–F** is actually the active species that undergoes C–C coupling, through migratory insertion, in an independent parallel path *before* undergoing protonation to give the carbene, which is an inactive species. Indeed, when carbene **2c** was reacted in the presence of 2 equiv of NHEt_2_, no formation of coupled product **3c** was observed even after 24 h at room temperature, indicating that, although NHEt_2_ can deprotonate intermediate **[A]**, the conditions are not basic enough to deprotonate carbene **3c**, which therefore accumulates as an inactive species.

In a separate experiment, isolated carbene **2c** was treated with the stronger base KOH in CDCl_3_ at 0 °C. The ^31^P­{^1^H} NMR spectrum shows the neat and complete transformation of the signal of **2c** (δ 88.5 ppm) into a new signal (δ 91.5 ppm) attributable to **C–F** within minutes (Figure 2, *left*). Although **C–F** was too reactive to be isolated in pure form, the subsequent rapid addition of NH_4_PF_6_ produced the complete reprotonation of **C–F** to give back the carbene **2c**. No other phosphorus-containing species were detected under these conditions. This experiment demonstrates that the carbene can be reversibly deprotonated and reprotonated by manipulating the pH. Importantly, while **2c** was found to be unreactive, the evolution of **C–F** to form **3c** was readily observed by ^31^P­{^1^H} NMR upon warming up to room temperature (about 30 min at room temperature).

**Figure 2:**
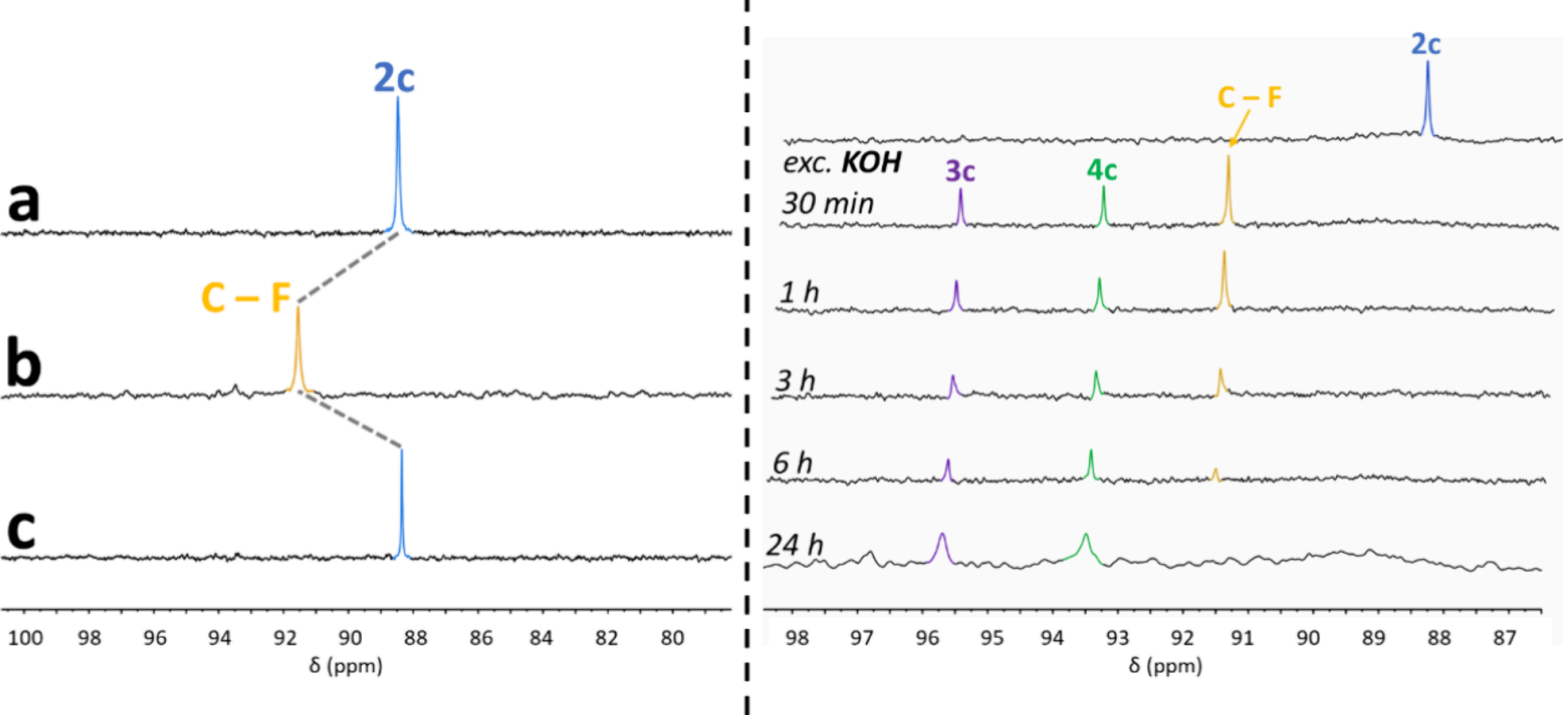
Left): ^31^P­{^1^H} NMR spectra (aliquot in CDCl_3_) showing the protonation/deprotonation experiment for compound **2c**. a) Spectrum of the isolated carbene. b) Spectrum of the deprotonated carbene **2c**(**C–F**) obtained 30 min after the addition of excess of KOH at 0 °C. c) Spectrum showing the reprotonation of **2c­(C–F)** to **2c** after adding NH_4_PF_6_. Right):^31^P­{^1^H} NMR monitoring of the reaction of carbene **2c** with KOH at room temperature. The initial deprotonation produces the formamidinyl complex **C–F**, which evolves into **4c** (first C–C coupling) and ultimately to **3c** (second C–C coupling).

In order to gain further insight into the reaction mechanism, carbene species **2c** was subjected to an *in situ* ^31^P­{^1^H} NMR study in CDCl_3_, in which the dithiophosphate ligand provides a convenient ^31^P­{^1^H} NMR handle for monitoring the reaction. We reasoned that the bulkier **2c** complex, which incorporates the bulkiest substituents among carbenes **2** in terms of both the ortho position isocyanide and the dithiophosphate ligand (i.e., R^1^ = ^i^Pr; R^2^ = ^i^Pr, *see* Scheme 3), would facilitate monitoring of the reaction by slowing the reaction rate and providing the possibility of kinetically stabilized reactive intermediates. Prior to the addition of KOH, only a singlet at δ 88.5 ppm characteristic of **2c** was observed in the ^31^P­{^1^H} NMR spectrum. Within 30 min of the addition of KOH at room temperature, complete deprotonation of **2c** took place, as shown by the disappearance of the signal at 88.6 ppm and the formation of **C–F** (δ 91.5 ppm). At this stage, a small amount of **3c** (δ 95.7 ppm), along with an additional species (δ 93.5 ppm), was also observed (Figure 2, *right*). Within hours, the signal corresponding to **C–F** completely disappeared, leading to the formation of **3c** and the additional species at δ 93.5 ppm. Although we note that this reaction is not optimized to obtain compound **3c**, which requires the coupling of three isocyanides, while starting complex **1c** only provides two isocyanides, the fact that the concentration of **3c** increases as the concentration of the species at δ 93.5 ppm decreases suggests that this species is an intermediate in route to **3c** (Figure 2, *right*).

We wondered whether the intermediate species at δ 93.5 ppm could be the species corresponding to the first C–C coupling (*see* Scheme 4), and whether it would be possible to isolate this reactive intermediate. It should be noted that this intermediate species is observed only in the formation of **3c.** The rest of the substrates do not appear to provide sufficient steric shielding to kinetically stabilize the corresponding species. This shows the interesting possibility of tuning, at least to some extent, the reactivity of the coordinated isocyanide by using increasingly bulky substituents.

Working under the appropriate conditions and low temperature (*T* = 0 °C), the reaction of freshly in situ-prepared complex **1c** with 1 equiv of NHEt_2_ along with 1 equiv of KOH permitted the isolation of compound **4c** as red crystals suitable for X-ray determination (*see* Experimental Section). As can be seen in Figure 3 *A*, in the structure of **4c**, the Ni atom is involved in a highly strained and reactive 4-member metallacycle (4-NiCy) resulting from the coupling of two isocyanides. This constitutes a rare example of a four-membered skeleton formed through the isocyanide insertion process.^37–39^ The space-filling representation in Figure 3 *B* illustrates the steric shielding provided by the use of isopropyl substituents in **4c** versus that of a hypothetical complex with Me groups instead of ^i^Pr. It is clear that the introduction of the isopropyl substituents on *both* the isocyanide and the dithiophosphate units sterically shields the metal center, making it less prone to engage in reactivity (i.e., hindering the approach of an additional isocyanide). The ^13^C NMR spectrum presents two quaternary carbon resonances corresponding to the two carbons in the 4-member metallacycle at 164.3 ppm (NiC) and 159.4 ppm (NC). The ^31^P­{^1^H} NMR spectrum of **4c** in CDCl_3_ displays a single resonance at 93.5, indicating that **4c** was the intermediate in route to **3c** observed in the previous study (*vide supra*).

**Figure 3:**
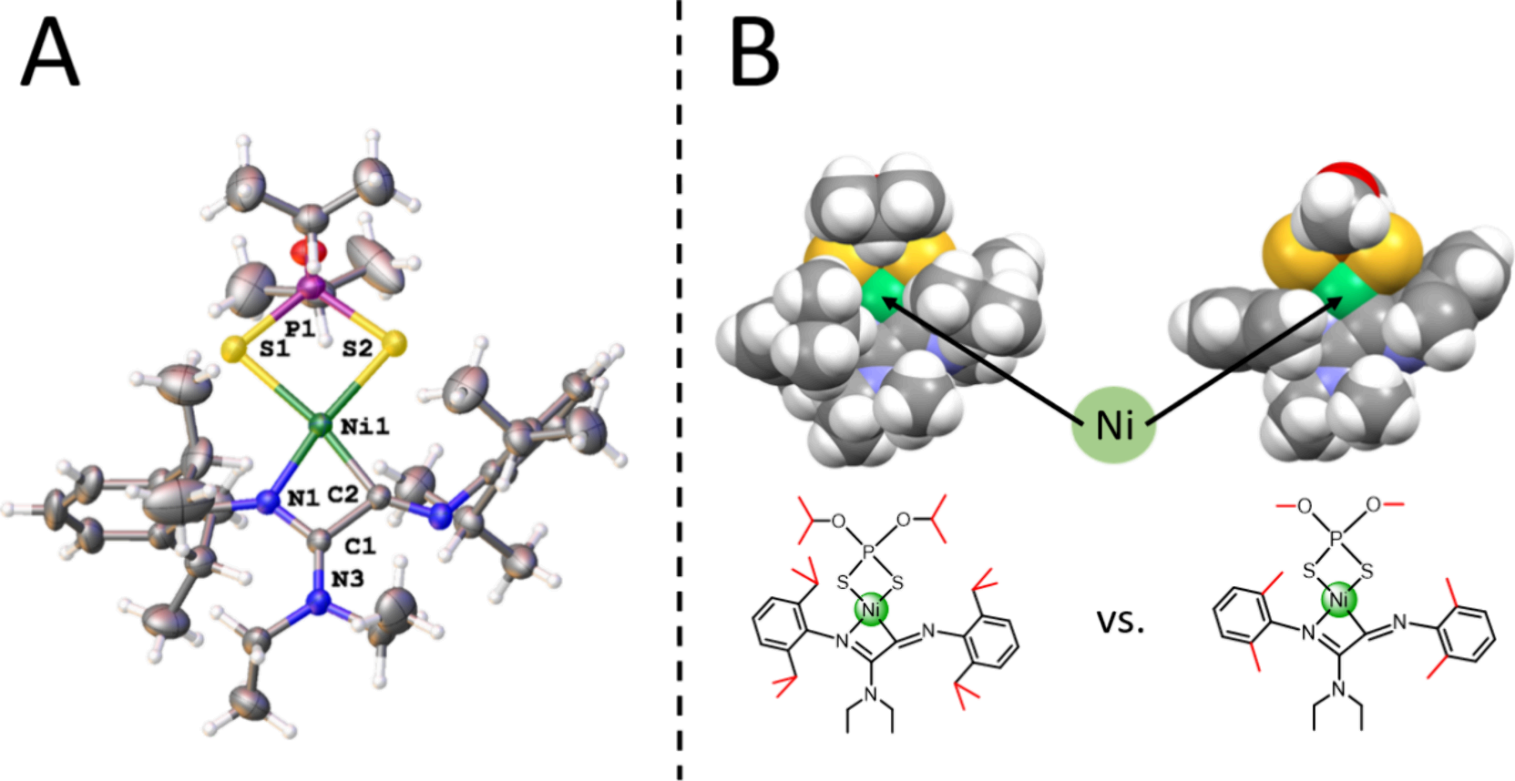
A) X-ray diffraction structure of complex **4c**. Selected bond lengths (Å) and angles (deg): Ni(1)–S(1): 2.3020(8), Ni(1)–S(2): 2.2187(9), Ni(1)–N(1): 1.894(2), Ni(1)–C(2): 1.880(3), S(1)–Ni(1)–S(2): 88.06(7), N(1)–Ni(1)–C(2): 70.35(11). B) Space-filling representation of 4-NiCy with different R substituents on the dithiophosphate ligand as well as the isocyanides: ^i^Pr vs Me. The structure of 4 Me-NiCy was simulated based on the experimental X-ray diffraction structure of **4c**.

To further prove that **4c** is an intermediate in the synthesis of **3c**, **4c** was dissolved in CH_2_Cl_2_ and one equivalent of isocyanide was added. After 2 h of stirring at room temperature, ^31^P­{^1^H} NMR monitoring showed complete conversion into **3c.** Complex **3c** is suggested to be a thermodynamic sink, which would explain why the formation of products containing three isocyanides is preferred, despite the fact that only two isocyanides are coordinated in the starting complexes **1**.

Our finding that **C–F** is the active species in the coupling process is also in good agreement with experimental results reported in polymerization systems other than the cationic merry-go-round process. These use catalysts such as CpNi­(PPh_3_)­R (R= alkyl, aryl)^40^ or [(π-allyl)­Ni­(OCOCF_3_)],^3,4,10,23^ which are usually formed “in situ” by oxidative addition to a Ni(0) isocyanide complex. In these systems, the polymerization is initiated by the migratory insertion of an anionic (X-type) ligand (alkyl, aryl, allyl) to an adjacent coordination-activated isocyanide. The polymerization is then produced by successive migratory insertion of the resulting anionic chain. There is also extensive literature on discrete isocyanide coupling with a variety of other groups, which are based on migratory insertions of anionic groups into isocyanide coordinated to metal.^41,42^ It is important to note that complex **1c** is stable in the presence of one equivalent of KOH and isocyanide. Therefore, the role of KOH is not to initiate the coupling mechanism by acting as an X-type ligand, but to provide a basic enough medium to form the key reactive **C–F** species.

It is worth noting here that couplings between palladium ADCs and coordinated isocyanides of a second Pd complex to give dinuclear Pd complexes have been reported.^43^ In this case, however, it is believed that the dinuclear product forms via deprotonation of the Pd-ADC followed by its coupling with a coordinated isocyanide from an (unreacted) second molecule of *cis*-[PdCl_2_(CNR)_2_] and the coordination of the second N center of the ADC to the Pd center of this second complex (as in this case, two N nucleophilic centers are present in the ADC).

Having established the key role of formamidinyl intermediates **C–F** in the promotion of C–C coupling, we were able to optimize the synthesis of complexes **3**. The use of a medium that was sufficiently basic to promote the formation of C–F while disfavoring inactive carbene **3** complexes was necessary. We found that the use of KOH (1 equiv) along with NHEt_2_ (1 equiv) provided complexes **3** in good isolated yields (30–60%) from complex **1** in the presence of one additional equivalent of the corresponding isocyanide (*see* Scheme 5 and Experimental Section). The reaction was monitored using IR spectroscopy. The lack of features in the 2165–2175 cm^–1^ region after 30 min indicated the insertion of all isocyanides. No carbene species were detected.

**Scheme 5:**
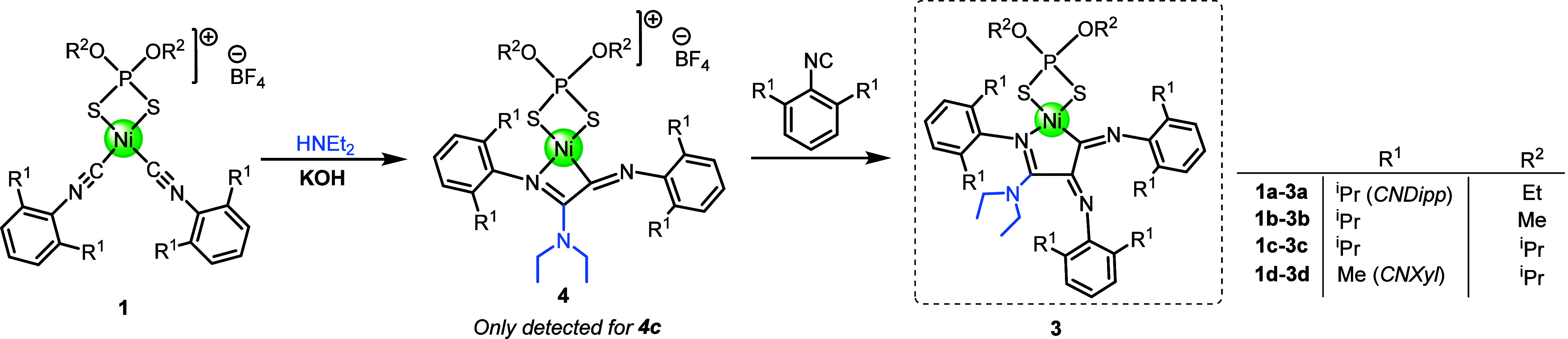
Optimized Reaction to Obtain Complexes **3**

Finally, as a supplement to this study, we decided to exploit the kinetic stabilization of intermediate **4c** and its tendency to react with additional isocyanide to relieve strain on the nickeliminoacyl bond to synthesize mixed-isocyanide species (*see* Scheme 6). Reaction of complex **1c** with NHEt_2_ (1 equiv) and one equivalent of KOH at 0 °C produced **4c** *in situ*, which was subsequently reacted with one equivalent of 2,6-dimethylphenylisocyanide in CH_2_Cl_2_ to afford **5** in 10% crystalline yield. Prior to the structural characterization of **5** by X-ray crystallography (Figure 4), it was characterized by spectroscopic and analytical techniques. Compound **5** presents a similar five-membered nickelmetallacycle (5-NiCy) to that observed for compounds **3**, but this metallacycle arises from the coupling of two 2,6-diisopropylphenyl isocyanides (CNDipp) and one 2,6-dimethylphenyl isocyanide (CNXyl). The carbon atom of the iminoacyl group derived from coupling of CNDipp forms part of 5-NiCy and is bound to the Ni­(II) center, while the nitrogen atom remains exocyclic. This indicates that the reactive species **4c** could be synthetically useful for the preparation of metallacycle nickel complexes derived from the insertion of two different isocyanides, as illustrated by the preparation of the mixed product **5**, which results from the insertion of two different isocyanides in a controlled, stepwise manner that resembles the first steps of a living polymerization of isocyanides.

**Scheme 6:**
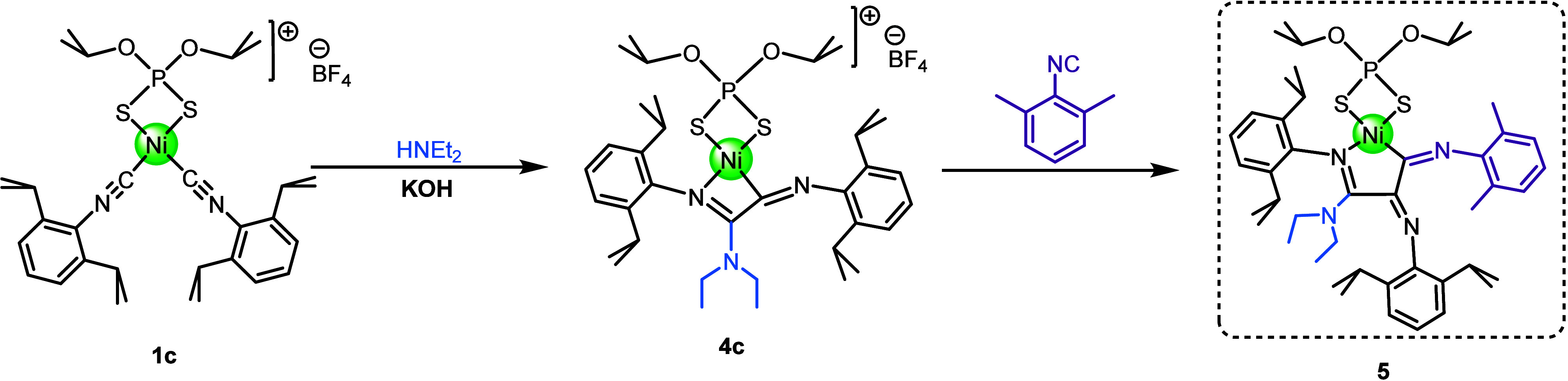
Synthesis of Mixed Compound **5**

**Figure 4:**
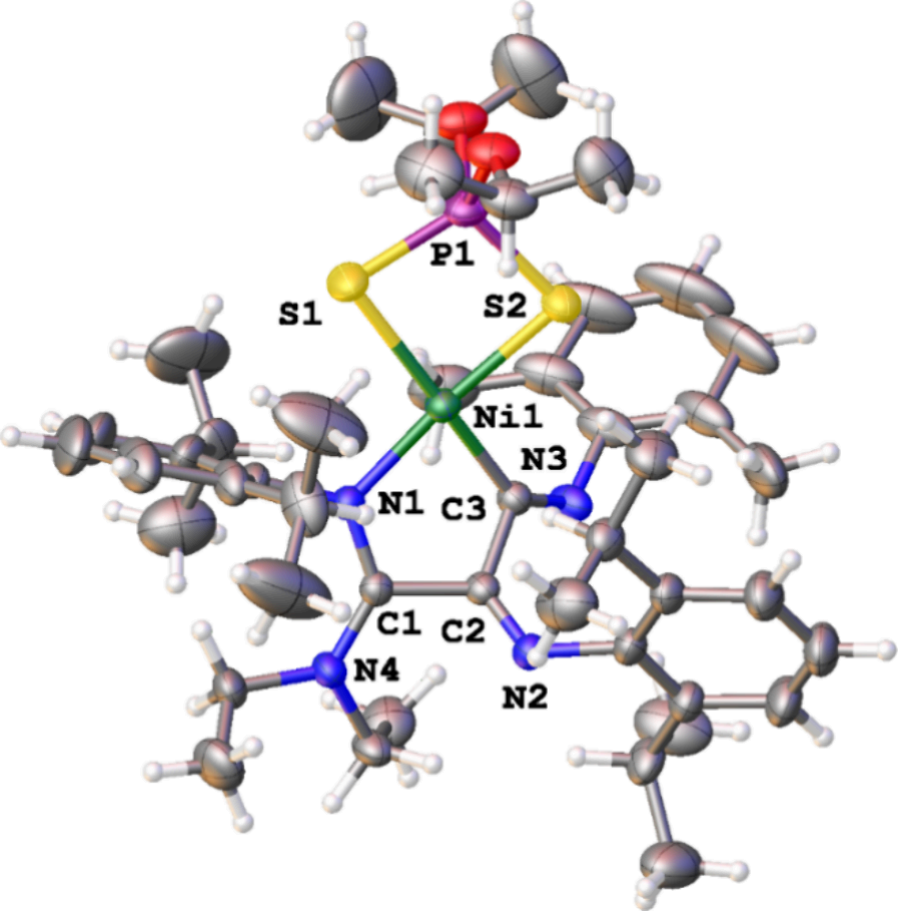
X-ray diffraction structure of complex **5**. Selected bond lengths (Å) and angles (deg): Ni(1)–S(1): 2.3181(12), Ni(1)–S(2): 2.2197(11), Ni(1)–N(1): 1.944(3), Ni(1)–C(3): 1.876(3), S(1)–Ni(1)–S(2): 87.17(4), N(1)–Ni(1)–C(3): 84.71(13).

## Conclusions

In the present work, we have identified side-products resulting from isocyanide coupling in the synthesis of cationic and bulky Ni-ADCs, and through study of the reaction, optimized their preparation and shed light on the mechanism by which these coupling products are formed. The use of steric hindrance at both the dialkyldithiophosphate and isocyanide ligands suppresses polymerization, giving rise to the formation of well-defined monomeric cationic Ni­(II) isocyanide complexes **3**. These products provide a “snapshot” of the initial steps of the polymerization mechanism via isocyanide coupling. Our results suggest that the active intermediate in the isocyanide coupling mechanism is the formamidinyl, not the carbene as was previously believed. We have also shown that by tuning the steric hindrance around the Ni atom through the substituents on the dithiophosphate ligands and the aryl ring of the isocyanide, it is possible to produce the C–C coupling in a step-by-step fashion, which enables the preparation of a rare mixed isocyanide Ni­(II) compound in a controlled manner. This illustrates that tuning of the reactivity of the system can be achieved by the judicious choice of the substituents at the dithiophosphate and isocyanide ligands.

These results should contribute to the rational design and development of well-defined Ni­(II)-based isocyanide complexes and are of great interest in light of the importance of polyisocyanide-based materials. We hope that this work will lead to a better understanding of isocyanide polymerization, allowing a greater degree of control in the design and synthesis of novel materials.

## Experimental Section

### General Experimental Techniques

All reagents were purchased from commercial suppliers and used without further purification. Compounds **1a**–**d** and **2a**–**d** were prepared according to a previously reported method.^33^ Solvents were used as received. Kieselguhr (diatomaceous earth, Merck, Germany) was used for filtration. NMR spectra were recorded using an Agilent DD2 500 MHz and an Agilent M2 400 MHz Agilent instrument, both equipped with a ONENMR probe in the NMR service of the Laboratory of Instrumental Techniques of the University of Valladolid (L.T.I., https://www.laboratoriotecnicasinstrumentales.es). ^1^H­{^13^C}, ^13^C and ^31^P­{^1^H} NMR chemical shifts (δ) are reported in parts per million (ppm) and are referenced to TMS, using solvents as internal references. Coupling constants (*J*) are reported in Hertz (Hz). Standard abbreviations are used to indicate multiplicity: s = singlet, d = doublet, t = triplet, m = multiplet. ^1^H, ^13^C and ^31^P assignments were performed using 2D NMR methods (gCOSY, gHSQCAD, and gHMBCAD). NMR spectra were analyzed using MNova v14.2.1. High-resolution mass spectra were recorded at the mass spectrometry service of the Laboratory of Instrumental Techniques of the University of Valladolid. Mass spectra were acquired using an Autoflex Speed mass spectrometer (Bruker Daltonics, Bremen, Germany) using a Smartbeam laser as the ionization source. The acceleration voltage was 20 kV in the reflection mode. HRMS spectra were analyzed using Bruker Data Analysis 4.1© (https://www.bruker.com).

### X-ray Diffraction Studies

Diffraction data were collected using an Oxford Diffraction Supernova diffractometer equipped with an Atlas CCD area detector and a four-circle κ goniometer. For the data collection, a Mo-microfocused source with multilayer optics was used. When necessary, crystals were mounted directly from solution using perfluorohydrocarbon oil to prevent atmospheric oxidation, hydrolysis, and solvent loss. Data integration, scaling, and empirical absorption correction were performed using the CrysAlisPro software package.^44^ The structure was solved by direct methods and refined by full-matrix least-squares against F2 with SHELX53 in OLEX2. Non-H atoms were refined anisotropically, and H atoms were placed at idealized positions and refined using the riding model.

#### Synthesis of **3a**

To a solution of **1a** (0.4 mmol, 282 mg) in 10 mL of dichloromethane, HNEt_2_ (0.4 mmol, 42 μL) and KOH (0.4 mmol, 22 mg) were added. The mixture was stirred for 5 min. CNDipp (0.4 mmol, 75 mg) was then added to the solution. The reaction was stirred for 90 min. Addition of hexane and slow evaporation at reduced pressure gave compound **3a** as a red precipitate. Yield: 260 mg, 37%. Crystals of **3a** suitable for X-ray analysis were grown in a saturated methanol solution at −25 °C. HR-MS (ESI-TOF, *m*/*z*); calcd. for C_47_H_71_N_4_NiNaO_2_PS_2_ = 899.4002; obtained = 899.4027 [M + Na]^+^. ^1^H NMR (500 MHz, Chloroform-d) δ 7.19–7.01 (7H, m, CH­(Ph, isocy)), 6.96–6.90 (1H, m, CH­(Ph, isocy), 6.88–6.81 (1H, m, CH­(Ph, isocy)), 4.01–3.87 (2H, m, (CH, ^i^Pr)), 3.86–3.75 (4H, m, H^6^), 3.54–3.43 (2H, m, (CH, ^i^Pr)), 3.42–3.33 (4H, m, H^4^), 3.29–3.16 (1H, m, (CH, ^i^Pr)), 3.07–2.99 (1H, m, (CH, ^i^Pr)), 1.60 (6H, d, *J* = 6.8 Hz, (CH_3_, ^i^Pr)), 1.42 (6H (CH_3_, ^i^Pr)), 1.36 (6H, d, *J* = 6.9 Hz, (CH_3_, ^i^Pr)), 1.28–1.21 (12H, m, (CH_3_, ^i^Pr)), 1.12 (6H, t, *J* = 7.1 Hz, H^7^), 1.06 (6H, t, *J* = 7.0 Hz, H^5^), 0.88–0.79 (6H, m, (CH_3_, ^i^Pr)). ^13^C NMR (126 MHz, Chloroform-d) δ 182.29 (1C, quaternary C), 160.77 (1C, quaternary C), 158.54 (1C, quaternary C), 146.60 (1C, C­(Ph, isocy)), 145.27 (1C, C­(Ph, isocy)), 144.62 (1C, C­(Ph, isocy)), 143.86 (2C, C­(Ph, isocy)), 140.94 (3C, C­(Ph, isocy)), 135.46 (1C, C­(Ph, isocy)), 126.78 (1C, CH­(Ph, isocy)), 123.85 (3C, CH­(Ph, isocy)) 122.96 (3C, CH­(Ph, isocy)), 121.09 (2C, CH­(Ph, isocy)), 62.61 (2C, C^6^), 43.88 (2C, C^4^), 29.33 (6C, (CH, ^i^Pr)), 24.43 (2C, (CH_3_, ^i^Pr)), 24.24 (4C, (CH_3_, ^i^Pr)), 24.09 (2C, (CH_3_, ^i^Pr)), 22.91 (4C, (CH_3_, ^i^Pr)), 14.04 (2C, C^5^). ^31^P­{^1^H} NMR (202 MHz, Chloroform-d) δ 97.12.

#### Synthesis of **3b**

To a solution of **1b** (0.4 mmol, 271 mg) in 10 mL of dichloromethane, HNEt_2_ (0.4 mmol, 42 μL) and KOH (0.4 mmol, 22 mg) were added. The mixture was stirred for 5 min. CNDipp (0.4 mmol, 75 mg) was then added to the solution. The reaction was stirred for 90 min. Addition of hexane and slow evaporation at reduced pressure gave compound **3b** as a red precipitate. Yield: 164 mg, 48%. Crystals of **3b** suitable for X-ray analysis were grown in a saturated methanol solution at −25 °C. HR-MS (ESI-TOF, *m*/*z*); calcd. for C_45_H_67_N_4_NiNaO_2_PS_2_ = 871.3689; obtained = 899.4027 [M + Na]^+^. ^1^H NMR (500 MHz, Chloroform-d) δ 7.18–7.03 (6H, m, CH­(Ph, isocy)), 6.98–6.93 (1H, t, *J* = 7.5 Hz, CH­(Ph, isocy)), 6.90–6.81 (2H, d, *J* = 7.2 Hz, CH­(Ph, isocy)), 4.00–3.82 (2H, m, (CH, ^i^Pr)), 3.51–3.37 (8H, m, 4H^4^ + 4H­(CH, ^i^Pr)), 1.61 (6H, d, *J* = 6.7 Hz, (CH_3_, ^i^Pr)), 1.59–1.49 (12H, m, 6H^6^ + 3H­(CH_3_, ^i^Pr)), 1.47–1.39 (6H, m, (CH_3_, ^i^Pr)), 1.36 (6H, d, *J* = 6.8 Hz, (CH_3_, ^i^Pr)), 1.25 (6H, d, *J* = 6.8 Hz, (CH_3_, ^i^Pr)), 1.06 (6H, q, *J* = 7.3 Hz, H^5^), 0.89–0.77 (3H, m, (CH_3_, ^i^Pr). ^13^C NMR (126 MHz, Chloroform-d) δ 181.84 (1C, quaternary C), 160.92 (1C, quaternary C), 158.73 (1C, quaternary C), 145.34 (1C, C­(Ph, isocy)), 144.83 (1C, C­(Ph, isocy), 144.13 (1C, C­(Ph, isocy)), 141.23 (3C, C­(Ph, isocy)), 135.74 (3C, C­(Ph, isocy)), 127.07 (1C, CH­(Ph, isocy)), 124.16 (2C, CH­(Ph, isocy)), 124.07 (1C, CH­(Ph, isocy)), 123.30 (1C, CH­(Ph, isocy)), 123.23 (2C, CH­(Ph, isocy)), 122.38 (2C, CH­(Ph, isocy)), 53.31 (2C, C^6^), 44.16 (2C, C^4^), 31.32 (2C, (CH, ^i^Pr)), 29.57 (2C, (CH, ^i^Pr)), 28.17 (2C, (CH, ^i^Pr)), 24.76 (2C, (CH_3_, ^i^Pr)), 24.47 (2C, (CH_3_, ^i^Pr)), 24.33 (3C, (CH_3_, ^i^Pr)), 23.24 (3C, (CH_3_, ^i^Pr)), 23.16 (2C, CH_3_, ^i^Pr), 14.29 (2C, C^5^). ^31^P­{^1^H} NMR (202 MHz, Chloroform-d) 101.71.

#### Synthesis of **3c**

To a solution of **1c** (0.4 mmol, 293 mg) in 10 mL of dichloromethane, HNEt_2_ (0.4 mmol, 42 μL) and KOH (0.4 mmol, 22 mg) were added. The mixture was stirred for 5 min. CNDipp (0.4 mmol, 75 mg) was then added to the solution. The reaction was stirred for 3 h. Addition of hexane and slow evaporation at reduced pressure gave compound **3c** as a red precipitate. Yield: 181 mg, 55%. ^1^H NMR (400 MHz, Chloroform-d) δ 7.17–7.02 (7H, m, CH­(Ph, isocy)), 6.94–6.80 (2H, m, CH­(Ph, isocy)), 4.59–4.43 (2H, m, H^6^), 3.96 (2H, m, (CH, ^i^Pr)), 3.41 (8H, m, 4H^4^, 4H­(CH, ^i^Pr)), 1.59 (6H, d, *J* = 6.8 Hz, (CH_3_, ^i^Pr)), 1.43 (6H, m, (CH_3_, ^i^Pr)), 1.36 (6H, d, *J* = 6.8 Hz, (CH_3_, ^i^Pr)), 1.25 (6H, d, *J* = 6.8 Hz, (CH_3_, ^i^Pr)), 1.11 (12H, d, *J* = 6.2 Hz, 6H^5^, 6H (CH_3_,^i^Pr)), 1.06 (12H, m, 12H^7^), 0.85 (6H, m, (CH_3_, ^i^Pr)). ^13^C NMR (101 MHz, Chloroform-d) δ 182.69 (1C, quaternary C), 161.12 (1C, quaternary C), 158.55 (1C, quaternary C), 145.25 (1C, C­(Ph, isocy)), 144.48 (1C, C­(Ph, isocy)), 143.64 (1C, C­(Ph, isocy)), 140.78 (3C, C­(Ph, isocy)), 135.30 (3C, C­(Ph, isocy)), 126.62 (1C, CH­(Ph, isocy)), 123.61 (3C, CH­(Ph, isocy)), 122.77 (3C, CH­(Ph, isocy)), 121.94 (2C, CH­(Ph, isocy)), 71.20 (2C, C^6^), 43.71 (2C, C^4^), 29.19 (3C, (CH, ^i^Pr)), 29.10 (3C, (CH, ^i^Pr)), 24.09 (2C, C^7^), 23.88 (2C, (CH_3_, ^i^Pr)), 23.53 (3C, (CH_3_, ^i^Pr)), 22.74 (1C, (CH_3_, ^i^Pr)), 13.86 (2C, C^5^).^31^P­{^1^H} NMR (202 MHz, Chloroform-d) 95.66.

#### Synthesis of **3d**

To a solution of **1d** (0.4 mmol, 249 mg) in 10 mL of dichloromethane, HNEt_2_ (0.4 mmol, 42 μL) and KOH (0.4 mmol, 22 mg) were added. The mixture was stirred for 5 min. CNXyl (0.4 mmol, 52 mg) was then added to the solution. The reaction was stirred for 30 min. Addition of hexane and slow evaporation at reduced pressure gave compound **3d** as a red precipitate. Yield: 92 mg, 31%. HR-MS (ESI-TOF, *m*/*z*); calcd. for C_37_H_52_N_4_NiNaO_2_PS_2_ = 759.2437; obtained = 759.2443 [M + Na]^+^. ^1^H NMR (400 MHz, Chloroform-d) δ 7.05–6.91 (5H, m, CH­(Ph, isocy)), 6.90–6.82 (1H, m, CH­(Ph, isocy)), 6.81–6.70 (3H, m, CH­(Ph, isocy)), 4.60–4.44 (2H, m, H^6^), 3.51–3.38 (4H, m, H^4^), 2.61 (12H, d, *J* = 18.0 Hz, H^7^), 1.89 (6H, s, (CH_3_, Ph)), 1.16 (12H, s, (CH_3_, Ph)), 1.05 (6H, t, *J* = 14.2 Hz, H^5^). ^13^C NMR (126 MHz, Chloroform-d) δ 187.54 (1C, quaternary C), 160.02 (1C, quaternary C), 156.69 (1C, quaternary C), 148.21 (2C, C­(Ph, isocy)), 147.61 (2C, C­(Ph, isocy)), 145.87 (2C, C­(Ph, isocy)), 131.34 (3C, C­(Ph, isocy)), 128.21 (1C, CH­(Ph, isocy)), 128.01 (1C, CH­(Ph, isocy)), 127.12 (2C, CH­(Ph, isocy)), 126.14 (1C, CH­(Ph, isocy)), 125.95 (1C, CH­(Ph, isocy)), 125.88 (1C, CH­(Ph, isocy)), 123.22 (1C, CH­(Ph, isocy)), 122.53 (1C, CH­(Ph, isocy)), 72.11 (2C, C^6^), 45.22 (2C, C^4^), 23.77 (4C, (CH_3_, Ph)), 19.78 (4C, C^7^), 18.69 (2C, (CH_3_, Ph)), 14.45 (2C, C^5^). ^31^P­{^1^H} NMR (202 MHz, Chloroform-d) δ 94.36.

#### Synthesis of **4c**

A solution of **1c** (0.4 mmol, 293 mg) in 10 mL of dichloromethane was placed in an ice bath. HNEt_2_ (0.4 mmol, 42 μL) and KOH (0.4 mmol, 22 mg) were added. The reaction was stirred for 1 h at 0 °C. The mixture was then filtered with Celite and dried by vacuum for 10 min. The residue was then redissolved in hexane, and some crystals were obtained through evaporation to give a red complex corresponding to compound **4c.** Yield: 34 mg, 12%. HR-MS (ESI-TOF, *m*/*z*); calcd. for C_36_H_58_N_3_NiO_2_PS_2_ = 718.3134; obtained = 718.3157 [M + H]^+^. ^1^H NMR (500 MHz, Chloroform-d) δ 7.14–7.08 (1H, m, CH­(Ph, isocy)), 7.07–7.00 (3H, m, CH­(Ph, isocy)), 6.97 (2H, m, CH­(Ph, isocy)), 4.67 (2H, m, H^5^), 3.87 (2H, m, (CH, ^i^Pr)), 3.46 (2H, m, (CH, ^i^Pr)), 1.61–1.51 (22H, m, 12H­(CH_3_, ^i^Pr); 6H^4^; 4H^3^)), 1.34 (6H, d, *J* = 6.9 Hz, (CH_3_, ^i^Pr)), 1.23–1.15 (18H, m, 6H­(CH_3_, ^i^Pr); 12H^6^). ^13^C NMR (126 MHz, Chloroform-d) δ 164.31 (1C, C^1^), 159.35 (1C, C^2^), 145.95 (1C, C­(Ph, isocy)), 141.35 (2C, C­(Ph, isocy)), 140.75 (2C, C­(Ph, isocy)), 135.82 (1C, C­(Ph, isocy)), 126.31 (1C, CH­(Ph, isocy)) 123.36 (2C, CH­(Ph, isocy)), 123.18 (1C, CH­(Ph, isocy)), 122.03 (2C, CH­(Ph, isocy)), 71.49 (2C, C^5^), 29.11­(2C, (CH, ^i^Pr)), 28.49 (2C, (CH, ^i^Pr)), 24.02 (6C, (CH_3_, ^i^Pr) and 2C^3^), 23.72 (4C, C^6^), 23.34 (2C, (CH_3_, ^i^Pr)), 23.19 (6C, (CH_3_, ^i^Pr) and 2C^4^), 23.08 (2C, (CH_3_, ^i^Pr)). ^31^P­{^1^H} NMR (202 MHz, Chloroform-d) 93.45.

#### Synthesis of **5**

To a solution of **1c** (0.4 mmol, 293 mg) in 10 mL of dichloromethane, HNEt_2_ (0.4 mmol, 42 μL) and KOH (0.4 mmol, 22 mg) were added. The mixture was stirred for 5 min. The reaction was stirred for 30 min. CNXyl (0.4 mmol, 52 mg) was then added to the solution. The mixture was stirred for 1 h. Addition of hexane and slow evaporation at reduced pressure gave compound **5** as a red precipitate. Yield: 24 mg, 7%. HR-MS (ESI-TOF, *m*/*z*); calcd. for C_45_H_68_N_4_NiO_2_PS_2_ = 849.38699; obtained = 849.3866 [M + H]^+^. ^1^H NMR (500 MHz, Chloroform-d) δ 7.19–6.97 (5H, m, CH­(Ph, isocy)), 7.04–6.99 (1H, m, CH­(Ph, isocy)), 6.79–6.69 (3H, m, CH­(Ph, isocy)), 4.55–4.40 (2H, m, H^6^), 3.94–3.78 (2H, m, (CH, ^i^Pr)), 3.69 (2H, m, (CH, ^i^Pr)), 3.49–3.33 (4H, m, H^4^), 2.01–1.73 (6H, m, (CH_3_, CNXyl)), 1.60 (d, *J* = 6.8 Hz, 6H­(CH_3_, ^i^Pr)), 1.44–1.37 (m, 6H (CH_3_, ^i^Pr)), 1.32 (d, *J* = 6.8 Hz, 6H (CH_3_, ^i^Pr)), 1.26 (6H, d, *J* = 6.8 Hz, (CH_3_, ^i^Pr)), 1.15–1.11 (6H, d, *J* = 6.4 Hz, H^7^), 1.10 (6H, d, *J* = 6.9 Hz, H^5^), 1.06–1.01 (6H, m, H^7^). ^13^C NMR (126 MHz, Chloroform-d) δ 185.77 (1C, quaternary C), 160.46 (1C, quaternary C), 157.83 (1C, quaternary C), 148.09 (1C, C­(Ph, isocy)), 145.01 (1C, C­(Ph, isocy)), 143.54 (1C, C­(Ph, isocy)), 140.62 (4C, C­(Ph, isocy)), 135.63 (2C, C­(Ph, isocy)), 126.87 (2C, CH­(Ph, isocy)), 126.58 (1C, CH­(Ph, isocy)), 123.66 (2C, CH­(Ph, isocy)), 123.57 (1C, CH­(Ph, isocy)), 122.70 (2C, CH­(Ph, isocy)), 121.99 (1C, CH­(Ph, isocy)), 71.45 (2C, C^6^), 43.96 (2C, C^4^), 29.23 (2C, (CH, ^i^Pr)), 28.97 (2C, (CH, ^i^Pr)), 24.21 (2C, (CH_3_, ^i^Pr)), 23.90 (4C, (CH_3_, ^i^Pr)), 23.62 (2C, C^7′^), 23.56 (2C, C^7^), 22.50 (2C, CH_3_, ^i^Pr), 18.10 (2C, CH_3_(CNXyl)), 14.08 (2C, C^5^).^31^P­{^1^NMR (202 MHz, Chloroform-d) δ 95.46.

## Supplementary Material



## Notes

NMR spectra, HRMS data, and X-ray data (PDF)
