# Supplementary material for: Snapshots of the Stopped Polymerization of a Hindered Isocyanide within the Coordination Sphere of Ni(II)
Source: Inorg Chem. 2025 Mar 7;64(11):5532–42. doi: 10.1021/acs.inorgchem.4c05461 (PMC12129260; doi:10.1021/acs.inorgchem.4c05461)
Supplement: Supplementary file 1 [file ic4c05461_si_001.pdf]

Supporting information

Snapshots of the stopped polymerization of a hindered isocyanide within the coordination sphere of Ni(II).

Clara del Carmen-Rodríguez,[a] *Lucía Álvarez-Miguel,*[b] *Celedonio M. Álvarez,*[a] *Raúl García-Rodríguez,*[a*] *Daniel Miguel*[a*].

[a] GIR MIOMET/IU CINQUIMA/Química Inorgánica, Facultad de Ciencias Universidad de Valladolid, E-47011 Spain.

Email: raul.garcia.rodriguez@uva.es; daniel.miguel@uva.es

[b] SOSCATCOM Group. Departamento de Química Orgánica y Química Inorgánica, Facultad de Farmacia and Instituto de Investigación Química "Andrés M. del Río" (IQAR) Universidad de Alcalá Campus Universitario, 28871 Alcalá de Henares, Madrid (Spain).

Contents

NMR studies and NMR spectra 4

^{31}P NMR monitoring reaction studies. 4

Figure S1. *$^{31}P\{^1H\}$ NMR spectra in $CDCl_3$ of carbene **2c** before (top) and after (bottom) 24 hours at reflux temperature in $CHCl_3$, showing that it remains stable under these conditions.* 4

Figure S2. *$^{31}P\{^1H\}$ NMR spectra in $CDCl_3$ of carbene **2c** before and after 2 h at 140 °C in a MW reactor in CH_2Cl_2 solution, showing that it remains stable under these conditions.* 4

Figure S3. *$^{31}P\{^1H\}$ NMR spectra of **1c** after the addition of various amounts of $HNEt_2$. (top) When 1 equivalent was added, only carbene **2c** was obtained in an incomplete reaction. Furthermore, the reaction was very slow. (bottom) When two equivalents of $HNEt_2$ were added, full and fast conversion of **1c** into **2c** was observed, along with the formation of small amounts of coupled compound **3c.** Note: A small amount of the intermediate **4c** was also observed.* 5

H, $^{13}C\{^1H\}$ and $^{31}P\{^1H\}$ NMR spectra. 6

Figure S4. *Compound **3a**, [Ni((SSP(OEt)$_{2)}$(CNDipp)$_3$(NEt$_2$)], with the atom labelling used in the NMR spectra.* 6

Figure S5. *^1H NMR spectrum of **3a** (500 MHz, $CDCl_3$).* 6

Figure S6. *$^{13}C\{^1H\}$ spectrum of **3a** (101 MHz, $CDCl_3$).* 7

Figure S7. *^1H–$^{13}C\{^1H\}$ HSQC NMR of compound **3a** (101 MHz, $CDCl_3$).* 7

Figure S8. *^1H–$^{13}C\{^1H\}$ HMBC NMR of compound **3a** (101 MHz, $CDCl_3$).* 8

Figure S9. *$^{31}P\{^1H\}$ spectrum of **3a** (200 MHz, $CDCl_3$).* 8

Figure S10. *Compound **3b**, [Ni((SSP(OMe)$_2$)(CNDipp)$_3$(NEt$_2$)], with the atom labelling used in the NMR spectra.* 9

Figure S11. *^1H NMR spectrum of **3b** (500 MHz, $CDCl_3$)..* 9

Figure S12. *$^{13}C\{^1H\}$ NMR spectrum of **3b** (101 MHz, $CDCl_3$)..* 10

Figure S13. *$^{31}P\{^1H\}$ NMR spectrum of **3b** (202 MHz, $CDCl_3$).* 10

Figure S14. *Compound **3c**, [Ni((SSP(OiPr)$_2$)(CNDipp)$_3$(NEt$_2$)], with the atom labelling used in the NMR spectra.* 11

Figure S15. *^1H NMR spectrum of **3c** (500 MHz, $CDCl_3$).* 11

Figure S16. *$^{13}C\{^1H\}$ spectrum of **3c** (101 MHz, $CDCl_3$).* 12

Figure S17. *^1H–$^{13}C\{^1H\}$ HSQC NMR of compound **3c** (101 MHz, $CDCl_3$).* 12

Figure S18. *^1H–$^{13}C\{^1H\}$ HMBC NMR of compound **3c** (101 MHz, $CDCl_3$).* 13

Figure S19. *$^{31}P\{^1H\}$ spectrum of **3c** (200 MHz, $CDCl_3$).* 13

Figure S20. *Compound **3d**, [Ni((SSP(O^{i}Pr)$_2$)(CNXyl)$_3$(NEt$_2$)], with the atom labelling used in the NMR spectra.* 14

Figure S21. *^1H NMR spectrum of **3d** (500 MHz, $CDCl_3$).* 14

Figure S22. *$^{13}C\{^1H\}$ NMR spectrum of **3d** (101 MHz, $CDCl_3$)* 15

Figure S23. *$^{31}P\{^1H\}$ NMR spectrum of **3d** (202 MHz, $CDCl_3$).* 15

Figure S24. *Compound **4c**, [Ni((SSP(O^{i}Pr)$_2$)(CNDipp)$_2$(NEt$_2$)], with the atom labelling used in the NMR spectra.* 16

Figure S25. ^{1}H NMR spectrum of **4c** (500 MHz, $CDCl_3$) 16
Figure S26. $^{13}C\{^{1}H\}$ spectrum of **4c** (101 MHz, $CDCl_3$). 17
Figure S27. ^{1}H–$^{13}C\{^{1}H\}$ HMBC NMR of compound **4c** (101 MHz, $CDCl_3$). 17
Figure S28. $^{31}P\{^{1}H\}$ spectrum of **4c** (200 MHz, $CDCl_3$). 18
Figure S29. Compound **5**, [Ni((SSP(O^iPr)$_2$)(CNXyl)(CNDipp)$_2$(NEt_2)], with the atom labelling used in the NMR spectra. 19
Figure S30. ^{1}H NMR spectrum of **5** (500 MHz, $CDCl_3$). 19
Figure S31. $^{13}C\{^{1}H\}$ NMR spectrum of **5** (101 MHz, $CDCl_3$). 20
Figure S32. ^{1}H–$^{13}C\{^{1}H\}$ HSQC NMR of compound **5** (101 MHz, $CDCl_3$). 20
Figure S33. ^{1}H–$^{13}C\{^{1}H\}$ HMBC NMR of compound **5** (101 MHz, $CDCl_3$). 21
Figure S34. $^{31}P\{^{1}H\}$ NMR spectrum of **5** (202 MHz, $CDCl_3$). 21
High resolution mass spectrometry data. 22
Figure S35. HR-MS (ESI-TOF) of **3a**. 22
Figure S36. HR-MS (ESI-TOF) of **3b**. 22
Figure S37. HR-MS (ESI-TOF) of **3d**. 23
Figure S38. HR-MS (ESI-TOF) of **4c**. 23
Figure S39. HR-MS (ESI-TOF) of **5**. 24
X-Ray Crystallographic Studies. 25
Figure S40. X-Ray diffraction structure of **3b** 25
Figure 41. X-Ray diffraction structure of **3c**. 25
Figure S42. X-Ray diffraction structure of **3d** 26
References 29

NMR studies and NMR spectra

^{31}P NMR monitoring reaction studies.

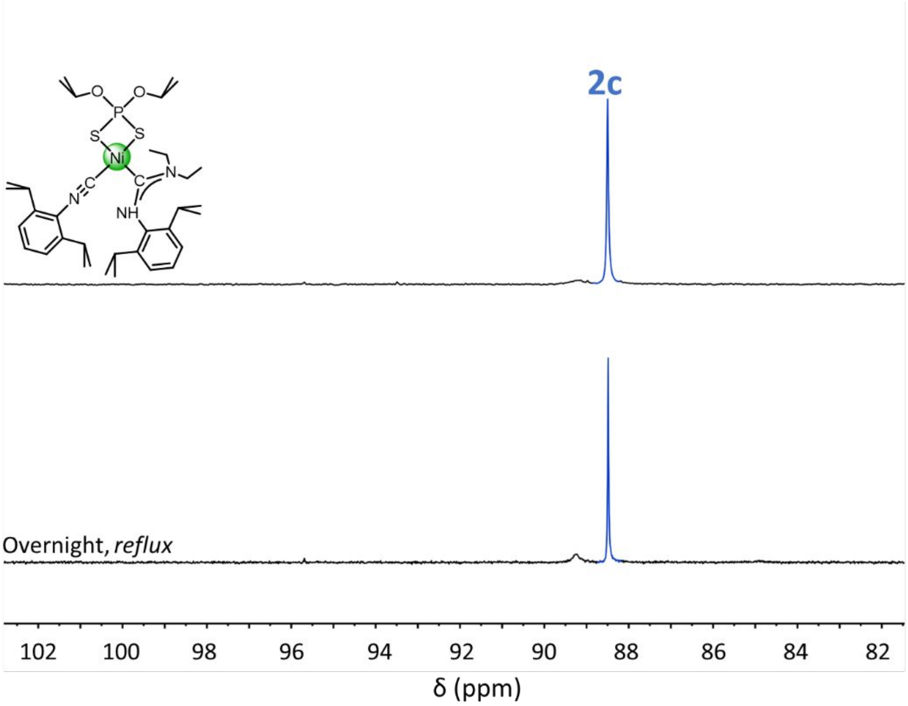

Figure S1. *${}^{31}P\{{}^{1}H\}$ NMR spectra in $CDCl_3$ of carbene* ***2c*** *before (top) and after (bottom) 24 hours at reflux temperature in $CHCl_3$, showing that it remains stable under these conditions.*

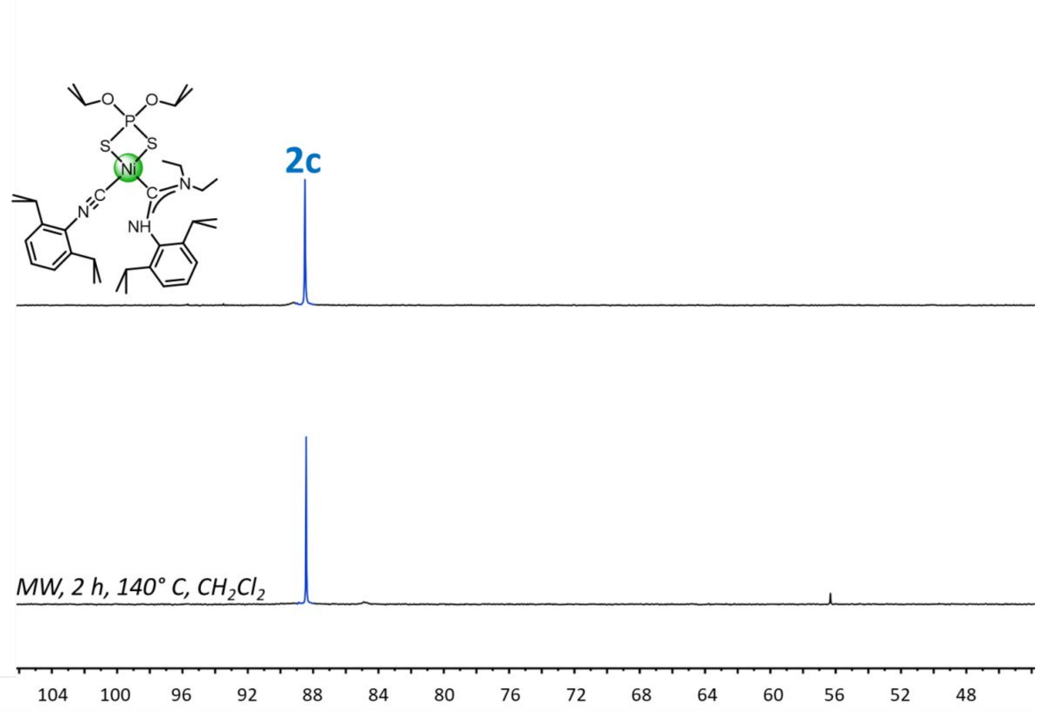

Figure S2. *${}^{31}P\{{}^{1}H\}$ NMR spectra in $CDCl_3$ of carbene* ***2c*** *before and after 2 h at 140 °C in a MW reactor in CH_2Cl_2 solution, showing that it remains stable under these conditions.*

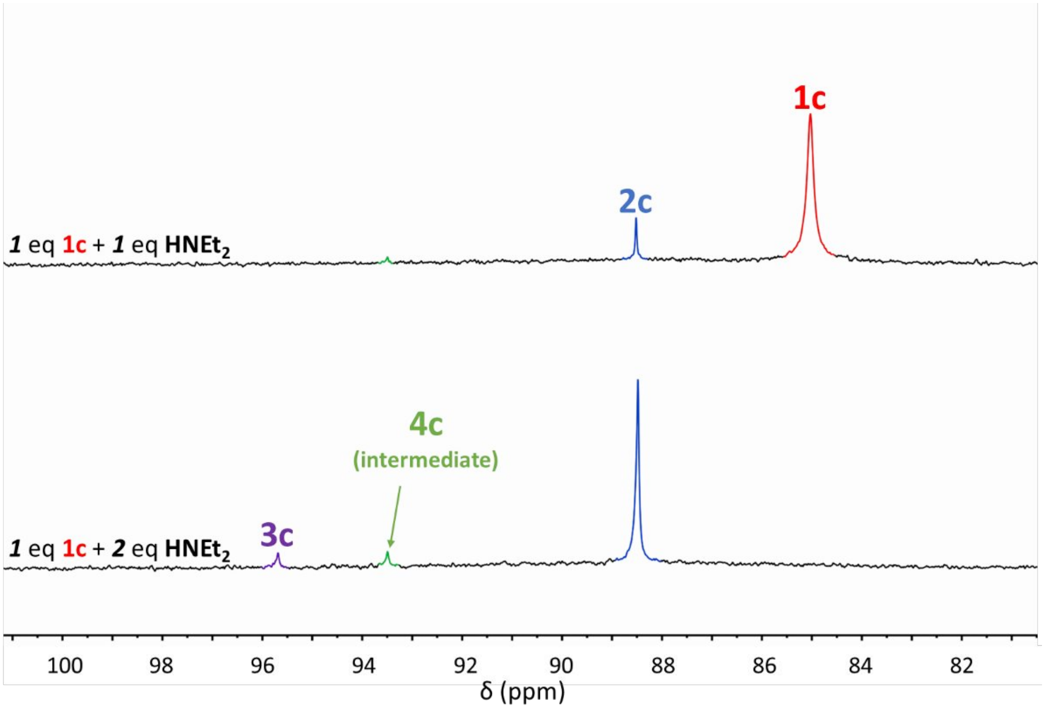

Figure S3. *^{31}P{^{1}H} NMR spectra of **1c** after the addition of various amounts of HNEt$_2$. (top) When 1 equivalent was added, only carbene **2c** was obtained in an incomplete reaction. Furthermore, the reaction was very slow. (bottom) When two equivalents of HNEt$_2$ were added, full and fast conversion of **1c** into **2c** was observed, along with the formation of small amounts of coupled compound **3c.** Note: A small amount of the intermediate **4c** was also observed.*

^{1}H, $^{13}C\{^{1}H\}$ and $^{31}P\{^{1}H\}$ NMR spectra.

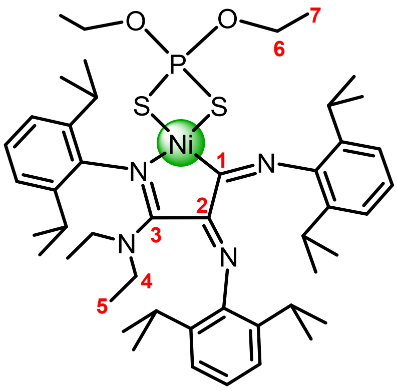

Figure S4. Compound **3a**, [Ni((SSP(OEt)2)(CNDipp)3(NEt2)], with the atom labelling used in the NMR spectra.

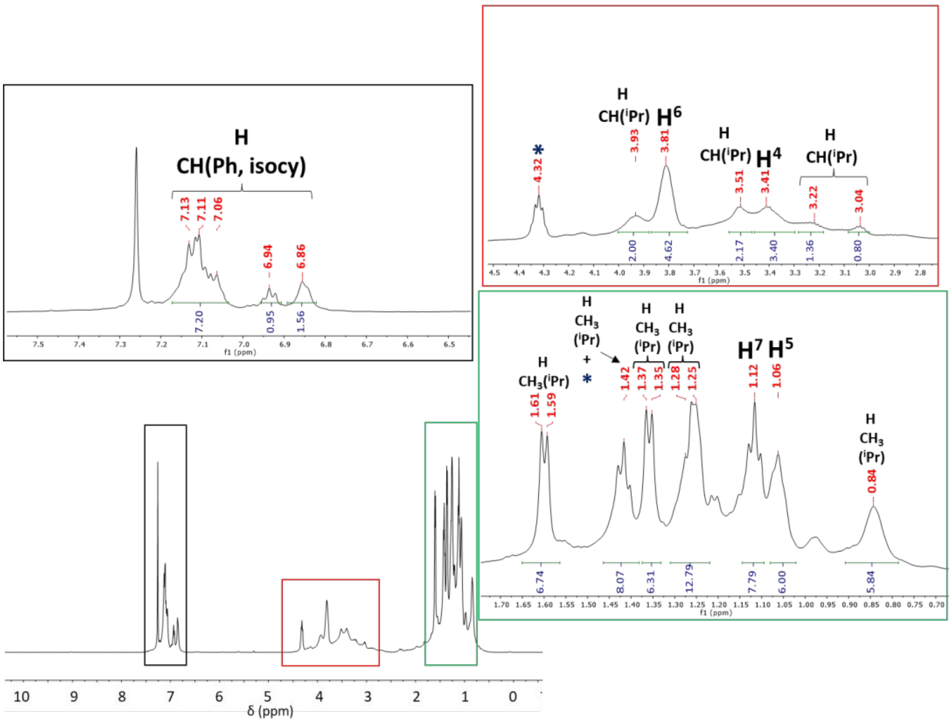

Figure S5. ^{1}H NMR spectrum of **3a** (500 MHz, $CDCl_3$). Signals marked with * correspond to the [Ni(SSP(OEt)2)2] complex generated in solution during the prolonged NMR experiment time.

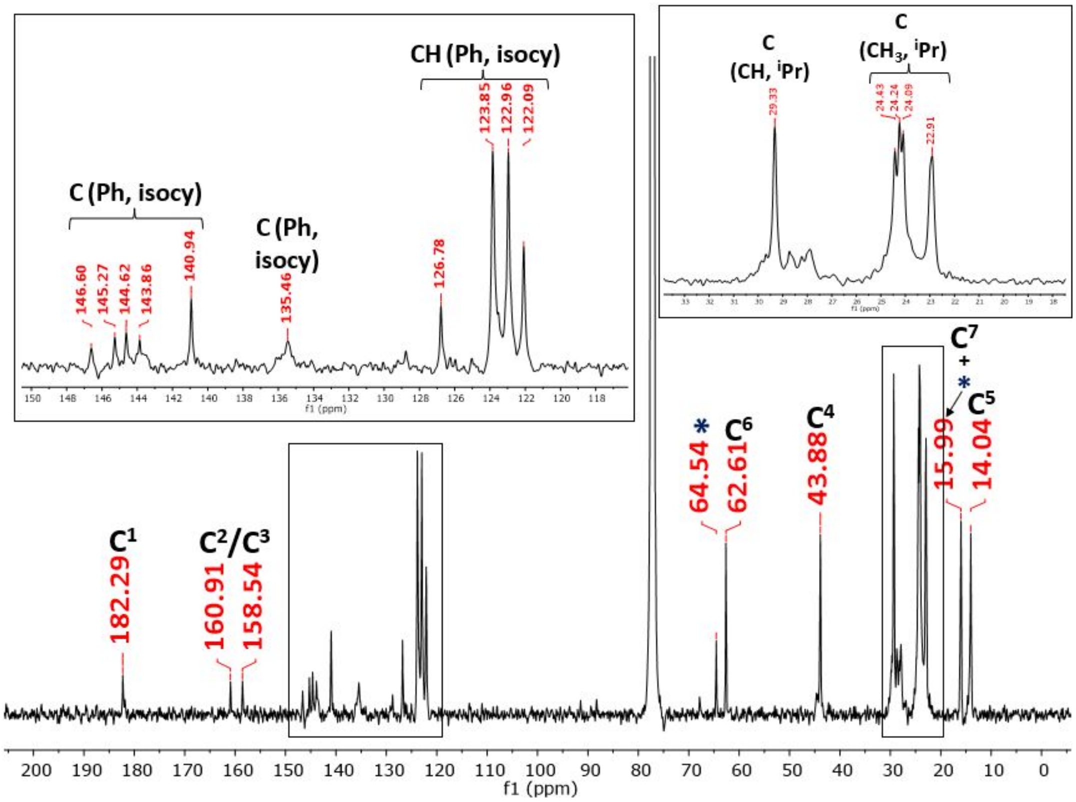

Figure S6. *$^{13}C\{^1H\}$ spectrum of **3a** (101 MHz, $CDCl_3$). Signals marked with * correspond to the $[Ni(S_2P(OEt)_2)_2]$ complex generated in solution during the prolonged NMR experiment time. The assignment of the three quaternary carbons as C1, C2 and C3 is tentative.*

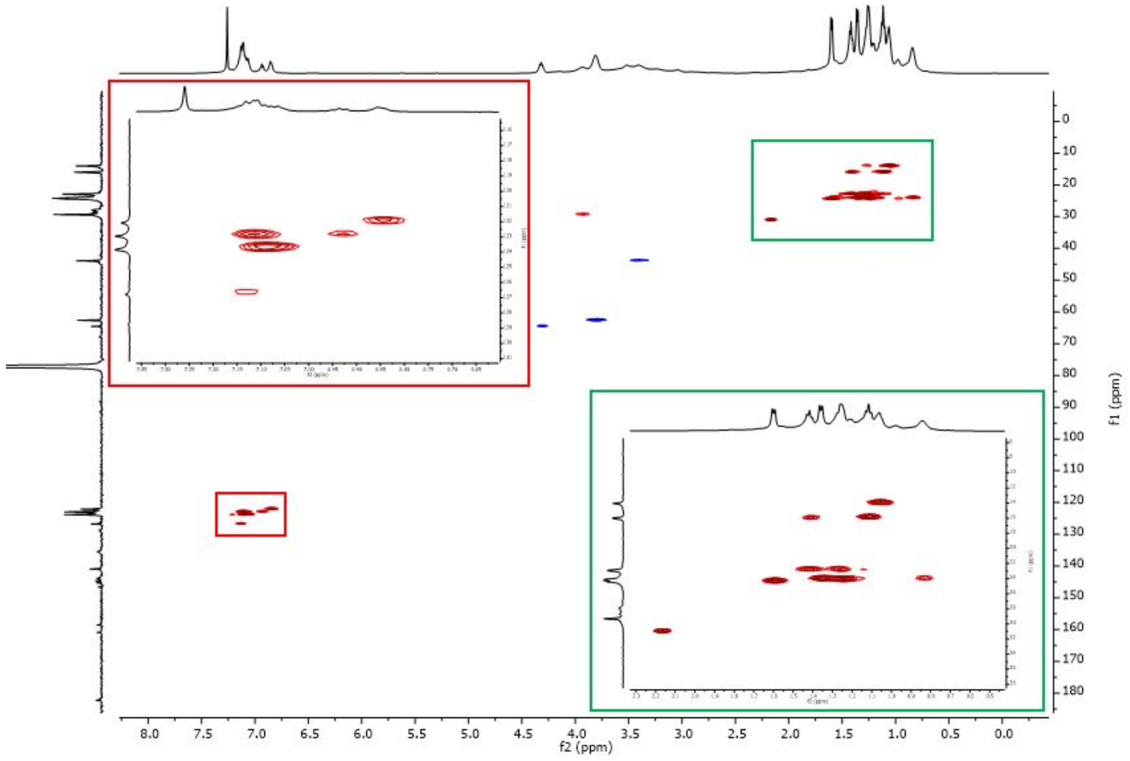

Figure S7. *^1H–$^{13}C\{^1H\}$ HSQC NMR of compound **3a** (101 MHz, $CDCl_3$).*

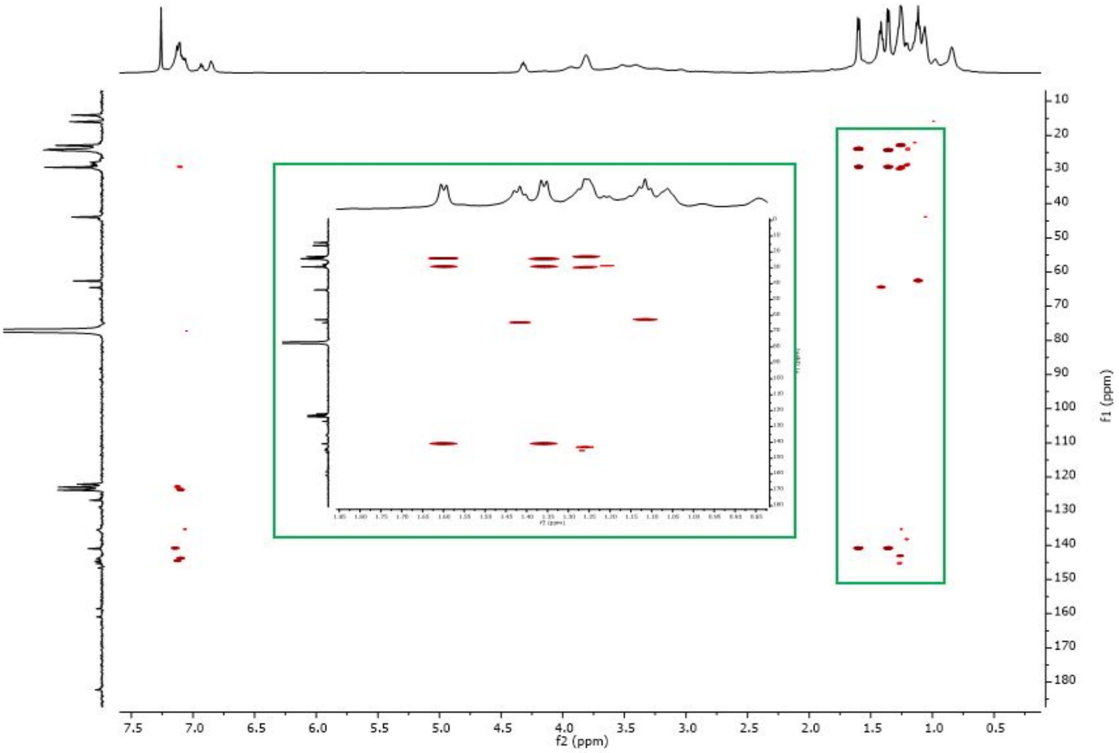

Figure S8. *^{1}H–$^{13}C\{^{1}H\}$ HMBC NMR of compound* ***3a*** *(101 MHz, $CDCl_3$).*

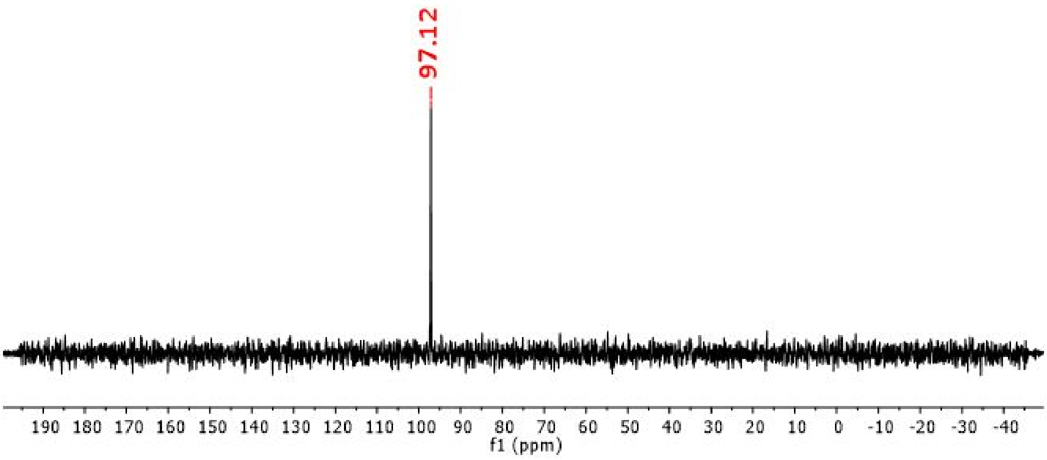

Figure S9. *$^{31}P\{^{1}H\}$ spectrum of* ***3a*** *(200 MHz, $CDCl_3$).*

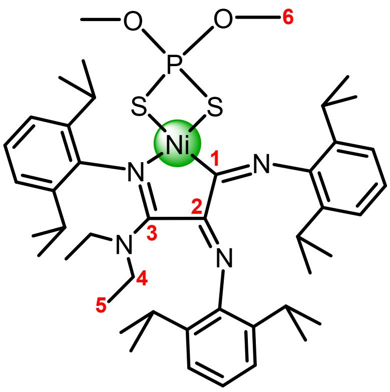

Figure S10. *Compound **3b**, [Ni((SSP(OMe)$_2$)(CNDipp)$_3$(NEt$_2$)], with the atom labelling used in the NMR spectra.*

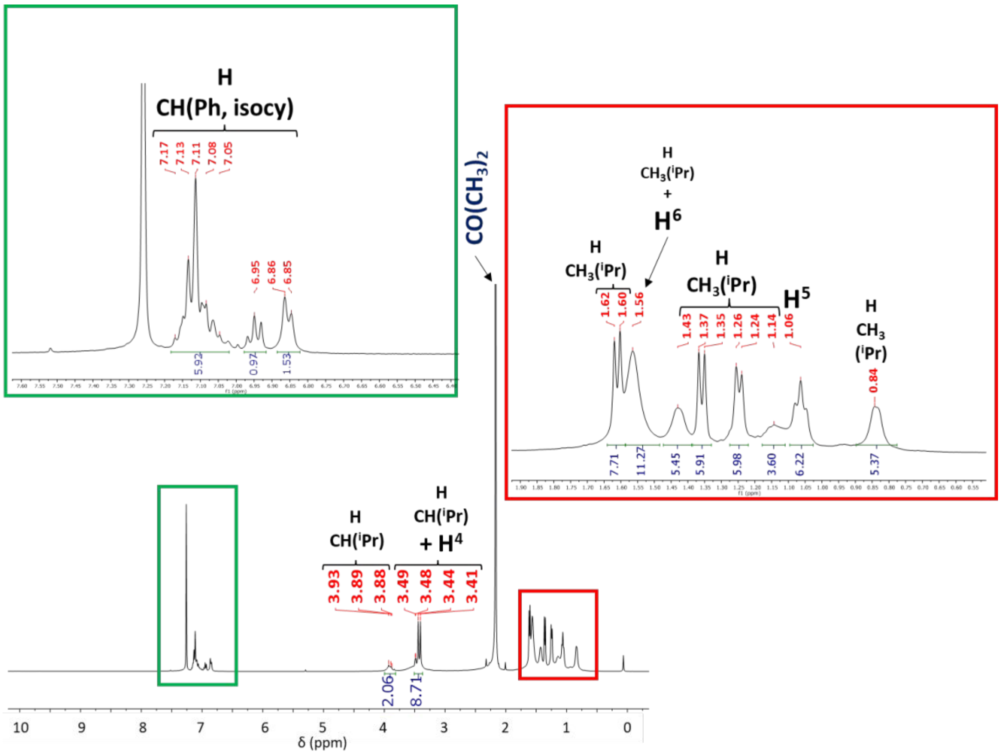

Figure S11. *^{1}H NMR spectrum of **3b** (500 MHz, CDCl$_3$). Note: Residual H$_2$O is observed at 1.56 ppm.*

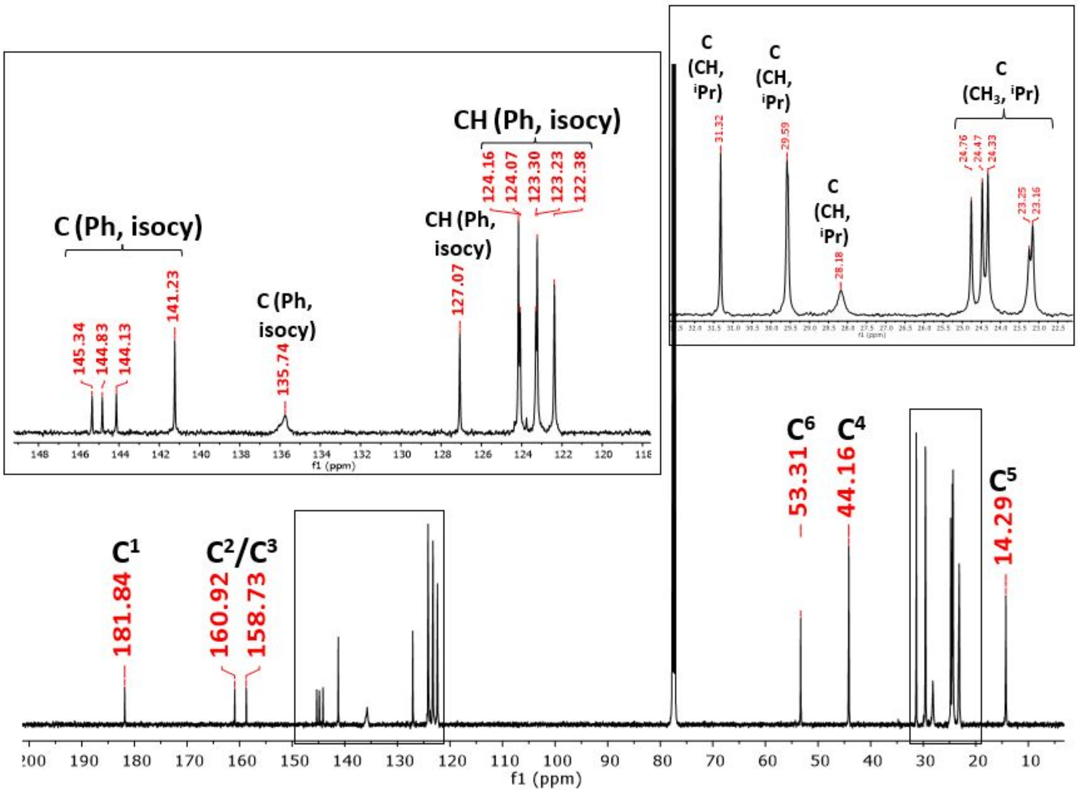

Figure S12. *$^{13}C\{^{1}H\}$ NMR spectrum of **3b** (101 MHz, $CDCl_3$). The assignment of the three quaternary carbons as C1, C2 and C3 is tentative.*

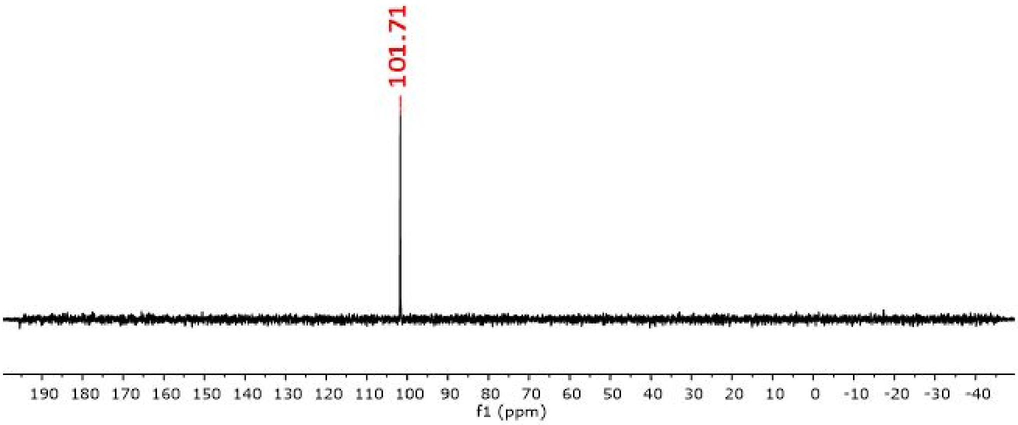

Figure S13. *$^{31}P\{^{1}H\}$ NMR spectrum of **3b** (202 MHz, $CDCl_3$).*

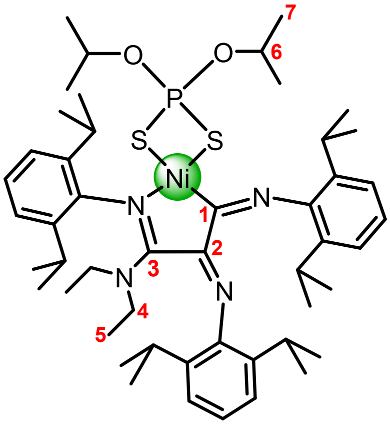

Figure S14. *Compound **3c**, [Ni((SSP(OiPr)$_2$)(CNDipp)$_3$(NEt$_2$)], with the atom labelling used in the NMR spectra.*

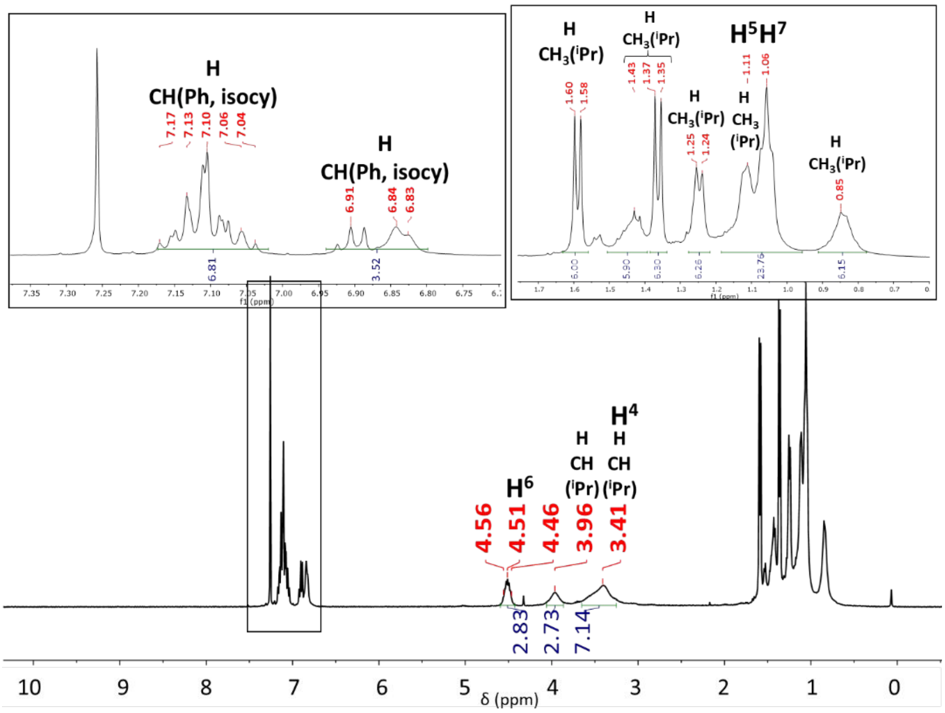

Figure S15. *^{1}H NMR spectrum of **3c** (500 MHz, CDCl$_3$).*

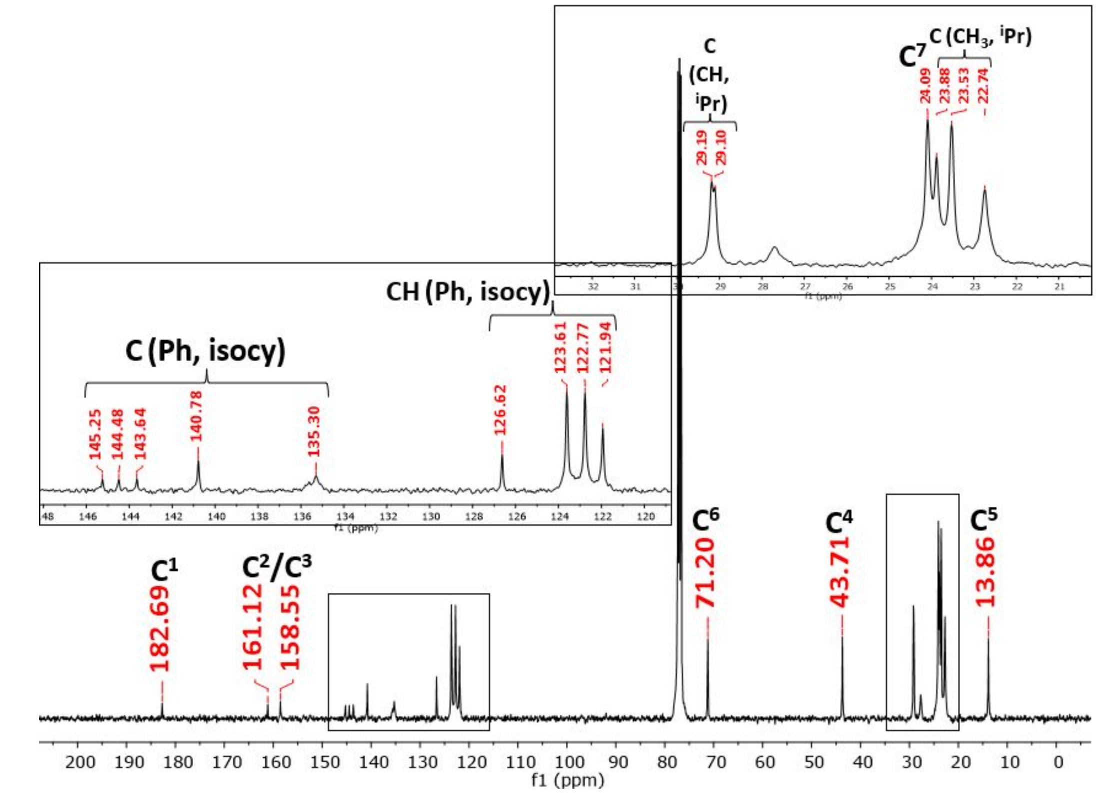

Figure S16. *$^{13}C\{^1H\}$ spectrum of **3c** (101 MHz, $CDCl_3$). The assignment of the three quaternary carbons as C1, C2 and C3 is tentative.*

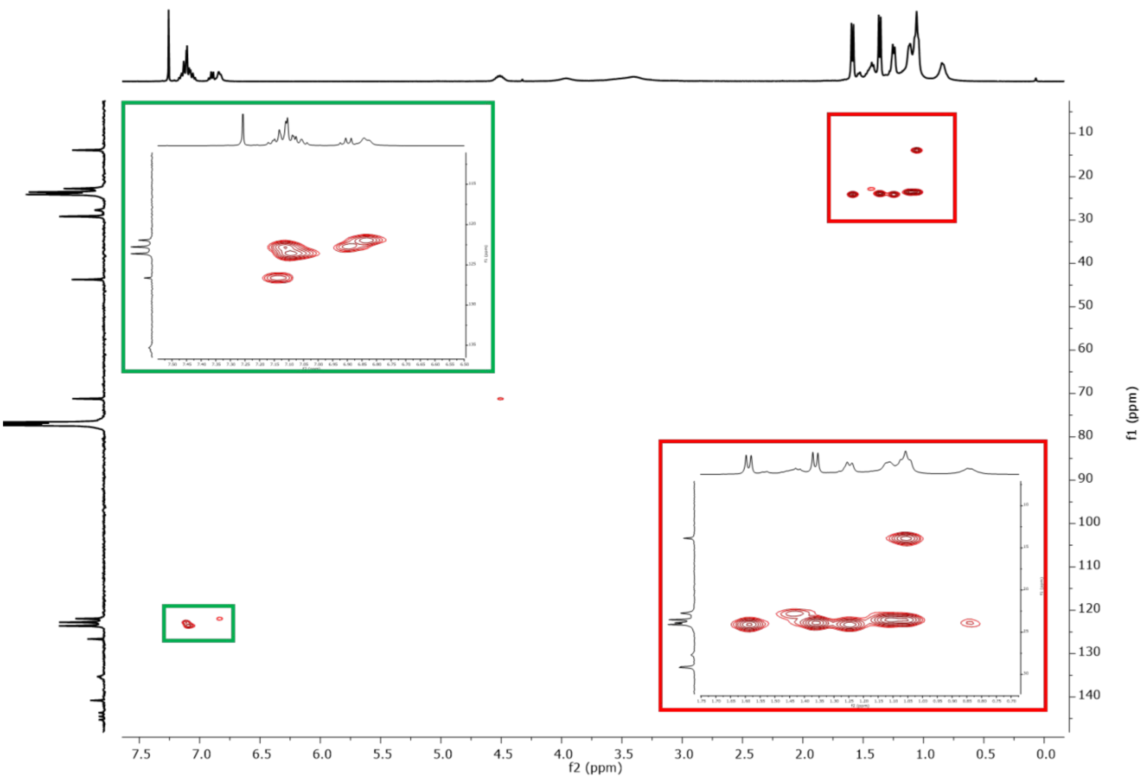

Figure S17. *^1H–$^{13}C\{^1H\}$ HSQC NMR of compound **3c** (101 MHz, $CDCl_3$).*

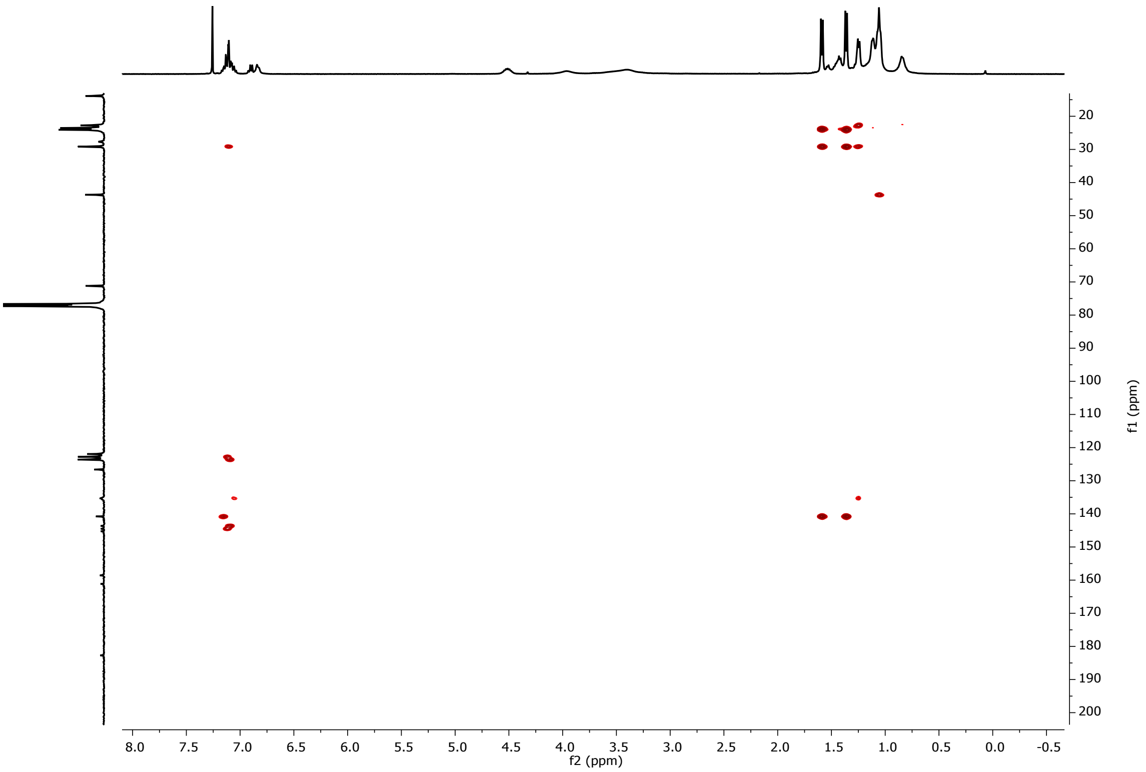

Figure S18. *^{1}H–$^{13}C\{^{1}H\}$ HMBC NMR of compound **3c** (101 MHz, $CDCl_3$).*

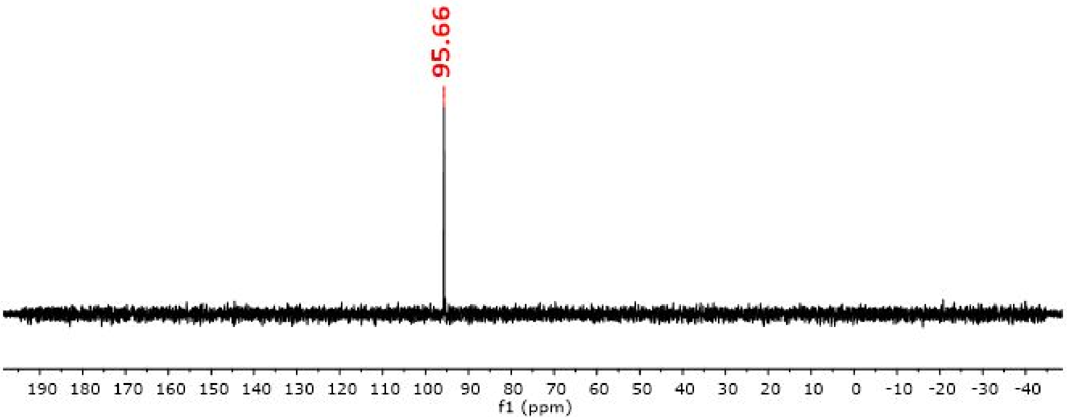

Figure S19. *$^{31}P\{^{1}H\}$ spectrum of **3c** (200 MHz, $CDCl_3$).*

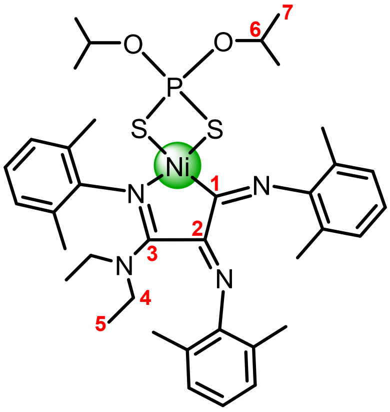

Figure S20. *Compound **3d**, [Ni((SSP(O^{i}Pr)$_2$)(CNXyl)$_3$(NEt$_2$)], with the atom labelling used in the NMR spectra.*

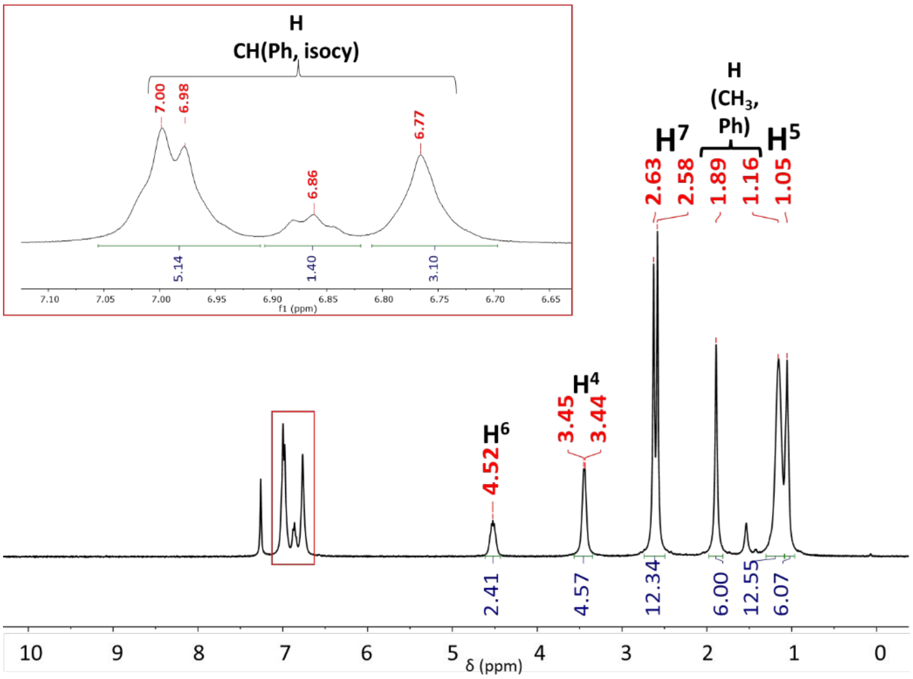

Figure S21. *^{1}H NMR spectrum of **3d** (500 MHz, CDCl$_3$).*

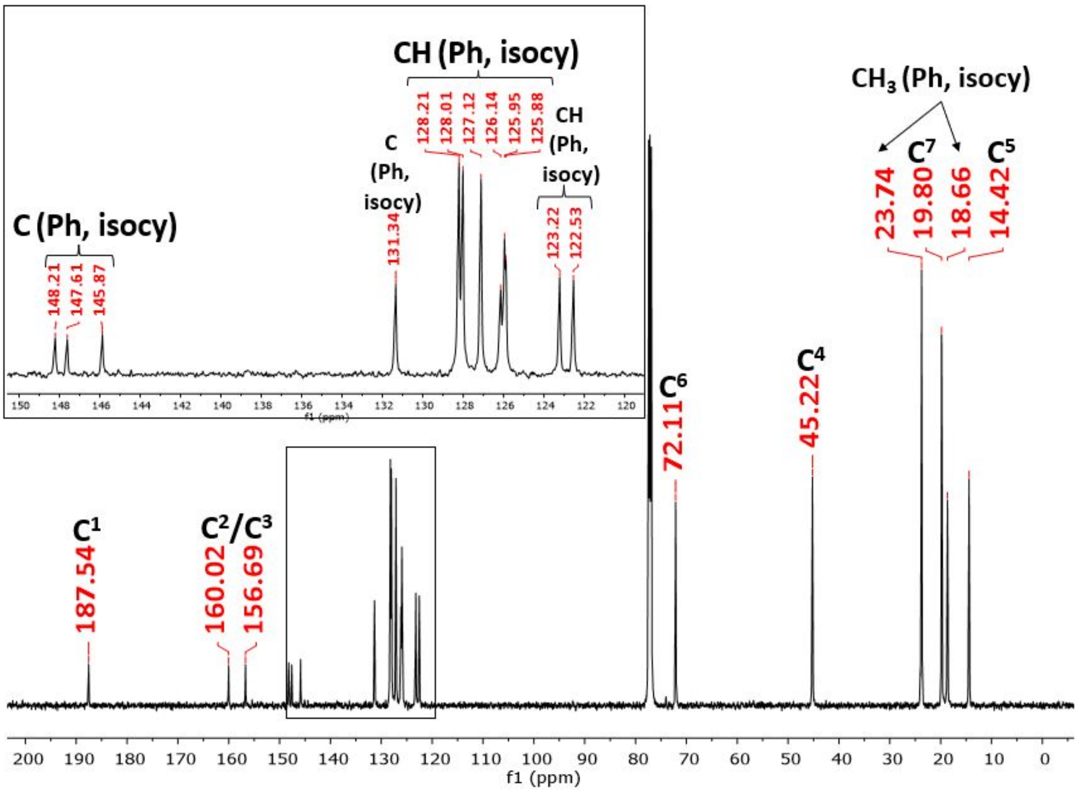

Figure S22. *$^{13}C\{^{1}H\}$ NMR spectrum of **3d** (101 MHz, $CDCl_3$). The assignment of the three quaternary carbons as C1, C2 and C3 is tentative.*

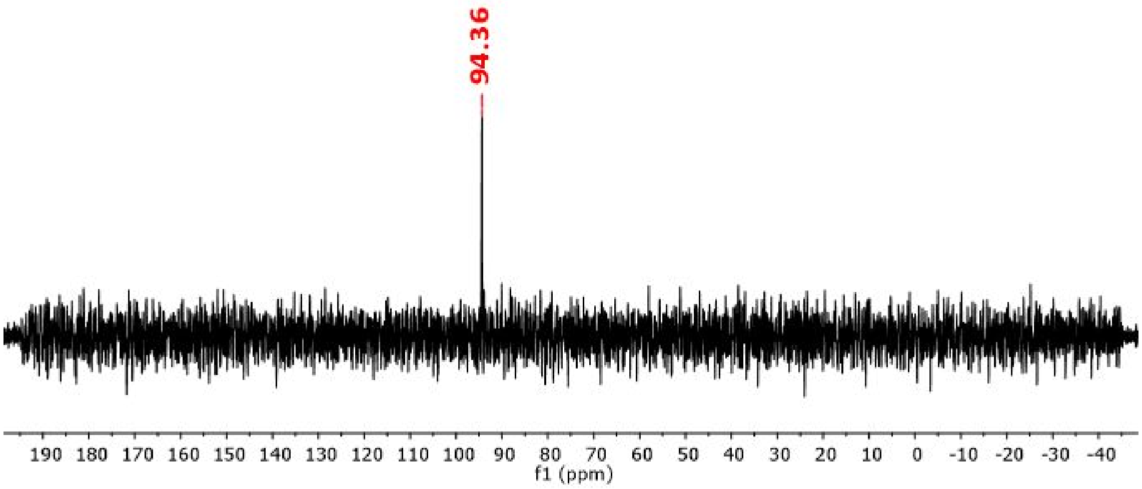

Figure S23. *$^{31}P\{^{1}H\}$ NMR spectrum of **3d** (202 MHz, $CDCl_3$).*

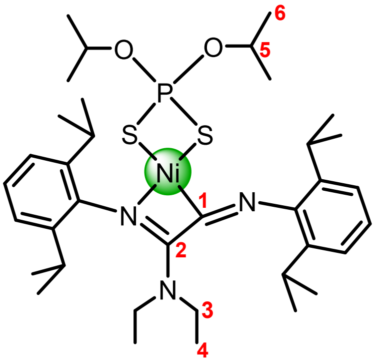

Figure S24. *Compound **4c**, [Ni((SSP(O^{i}Pr)$_2$)(CNDipp)$_2$(NEt$_2$)], with the atom labelling used in the NMR spectra.*

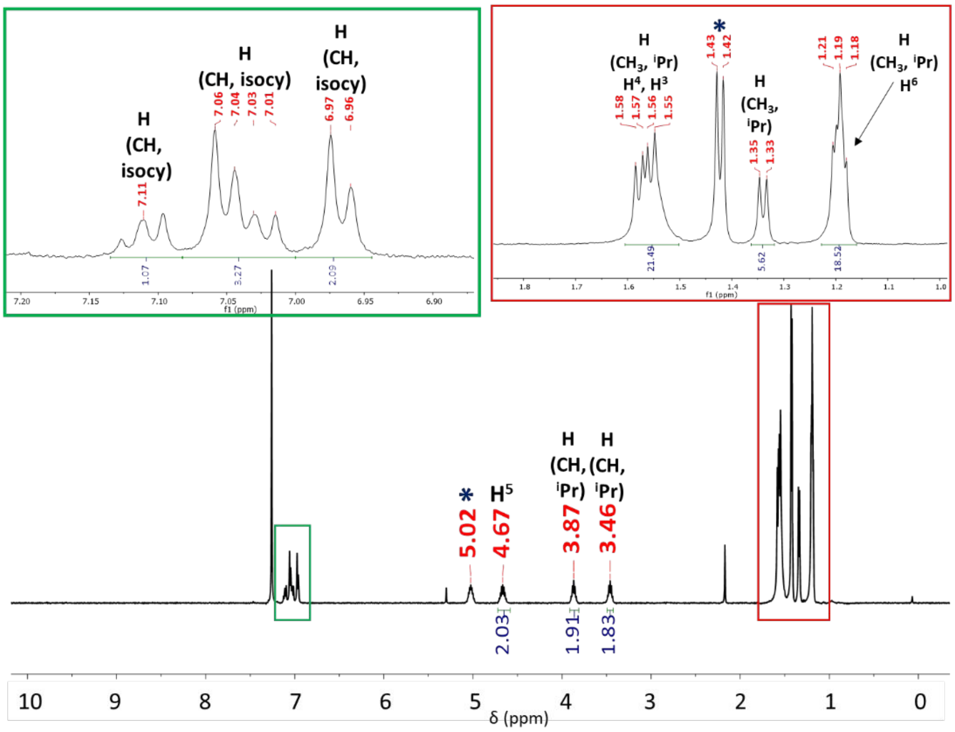

Figure S25. *^{1}H NMR spectrum of **4c** (500 MHz, CDCl$_3$). Signals marked with * correspond to [Ni(S$_2$P(O^{i}Pr)$_2$)$_2$], which was formed in situ during the experiment.*

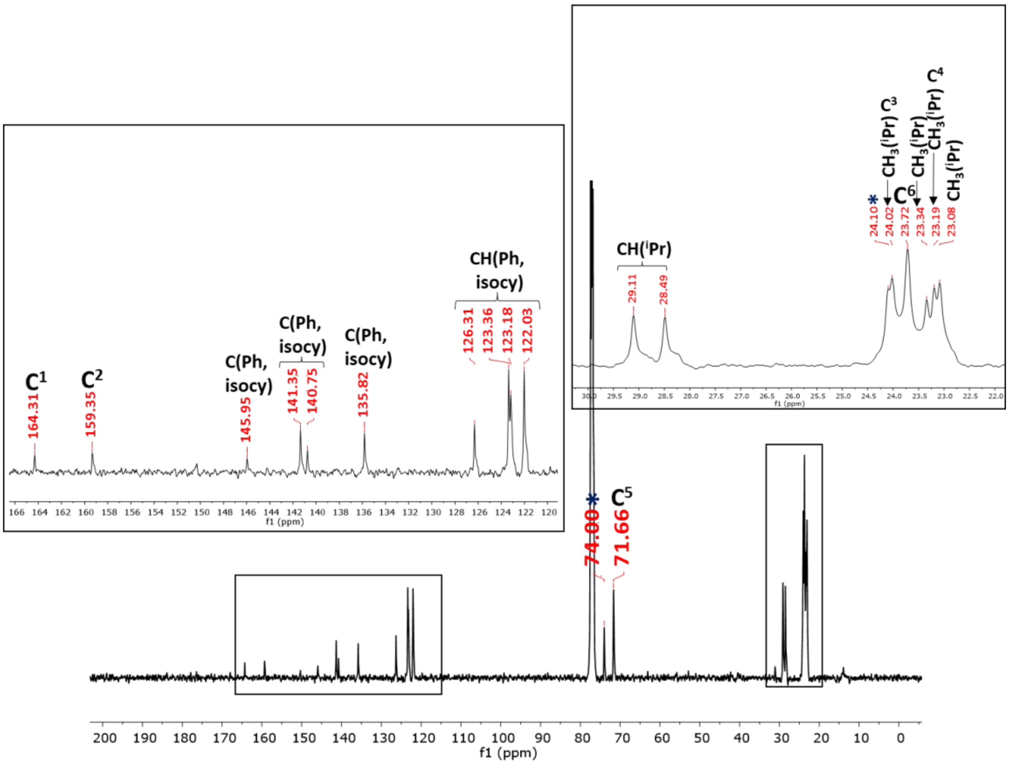

Figure S26. *$^{13}C\{^1H\}$ spectrum of **4c** (101 MHz, $CDCl_3$). The assignment of the two quaternary carbons as C1 and C2 is tentative.*

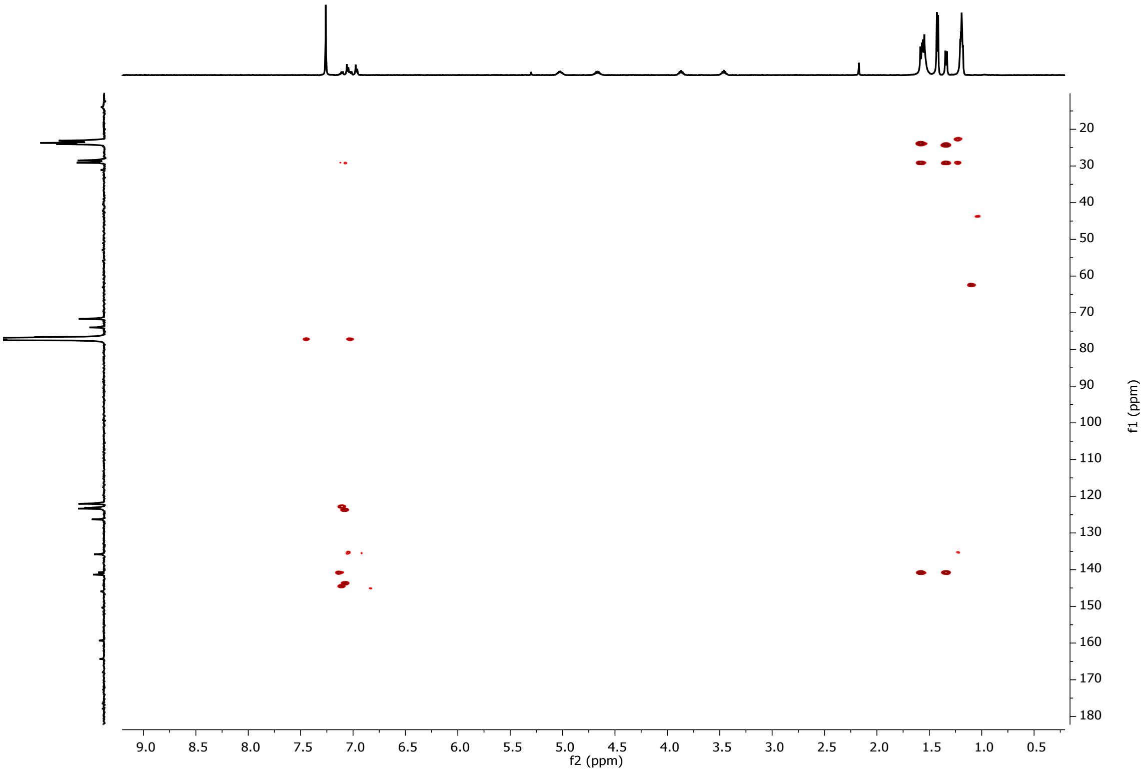

Figure S27. *^1H–$^{13}C\{^1H\}$ HMBC NMR of compound **4c** (101 MHz, $CDCl_3$).*

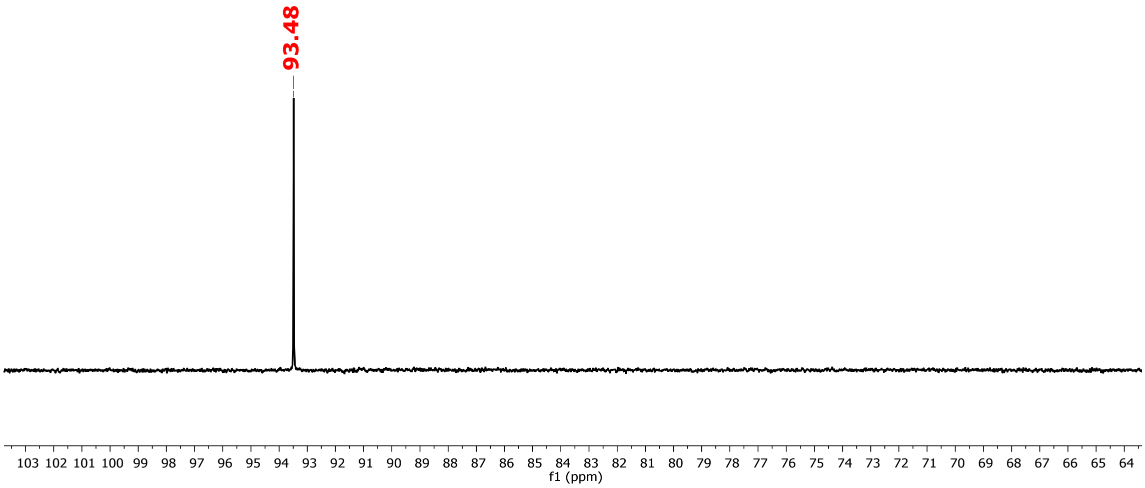

Figure S28. *$^{31}P\{^1H\}$ spectrum of* ***4c*** *(200 MHz, $CDCl_3$).*

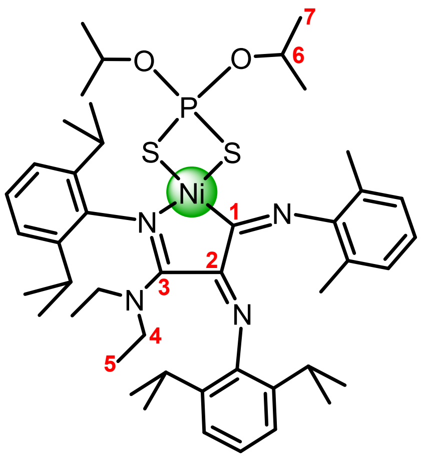

***Figure S29**. Compound **5**, [Ni((SSP(O^{i}Pr)$_2$)(CNXyl)(CNDipp)$_2$(NEt$_2$)], with the atom labelling used in the NMR spectra.*

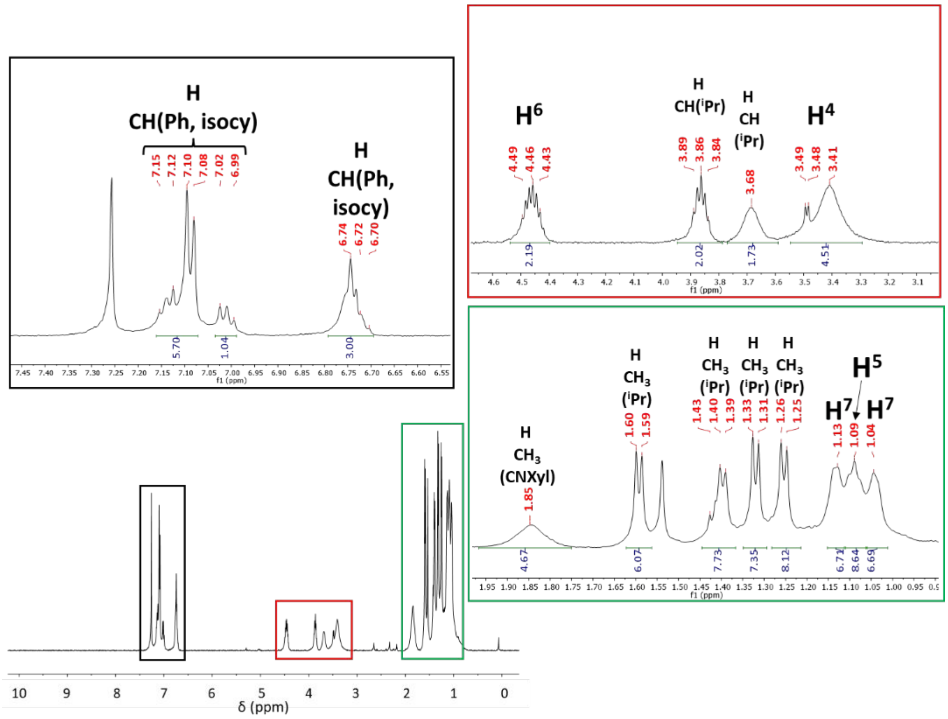

***Figure S30**. ^{1}H NMR spectrum of **5** (500 MHz, CDCl$_3$).*

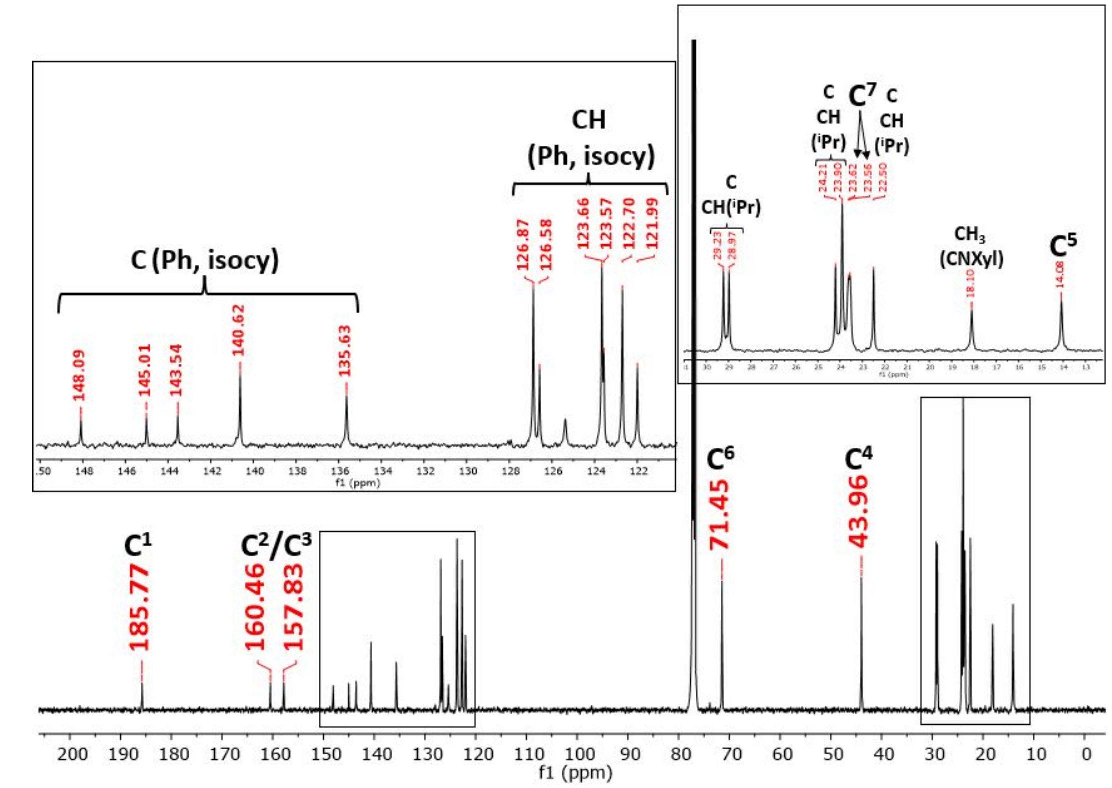

Figure S31. *^{13}C{^{1}H} NMR spectrum of **5** (101 MHz, $CDCl_3$). The assignment of the three quaternary carbons as C1, C2 and C3 is tentative.*

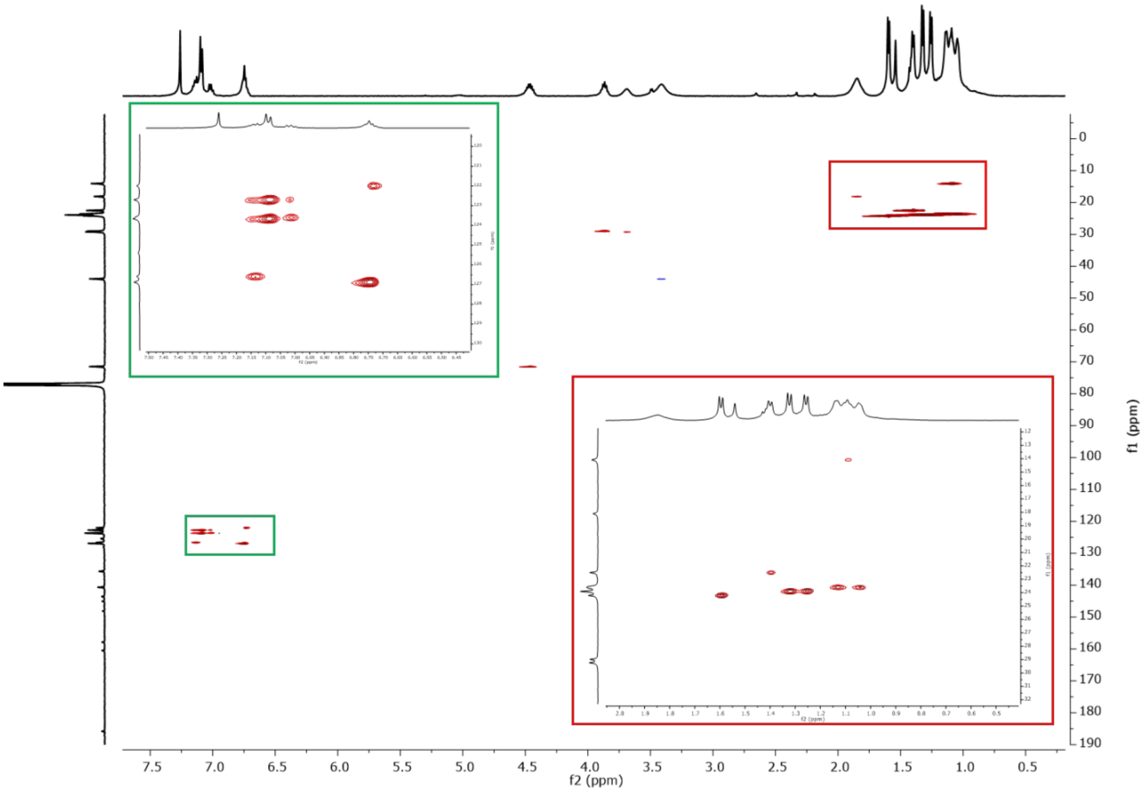

Figure S32. *^{1}H–^{13}C{^{1}H} HSQC NMR of compound **5** (101 MHz, $CDCl_3$).*

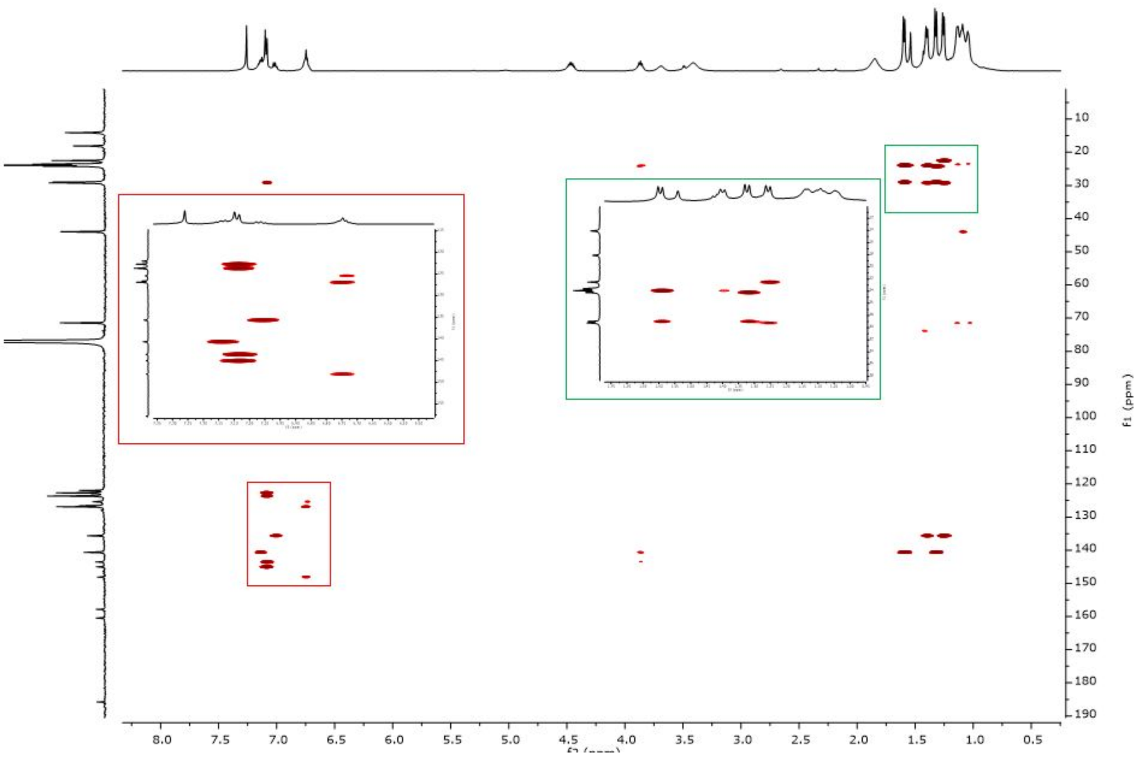

Figure S33. *^{1}H–$^{13}C\{^{1}H\}$ HMBC NMR of compound* ***5*** *(101 MHz, $CDCl_3$).*

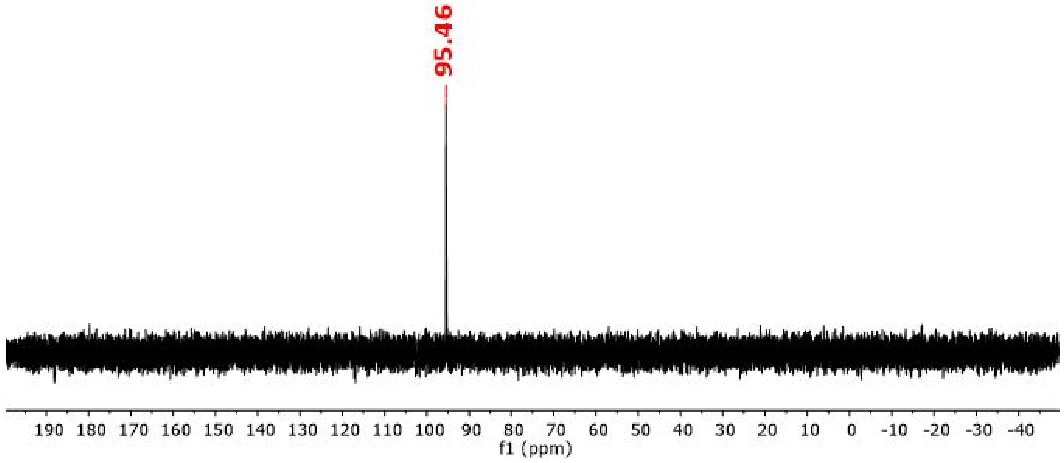

Figure S34. *$^{31}P\{^{1}H\}$ NMR spectrum of* ***5*** *(202 MHz, $CDCl_3$).*

High resolution mass spectrometry data.

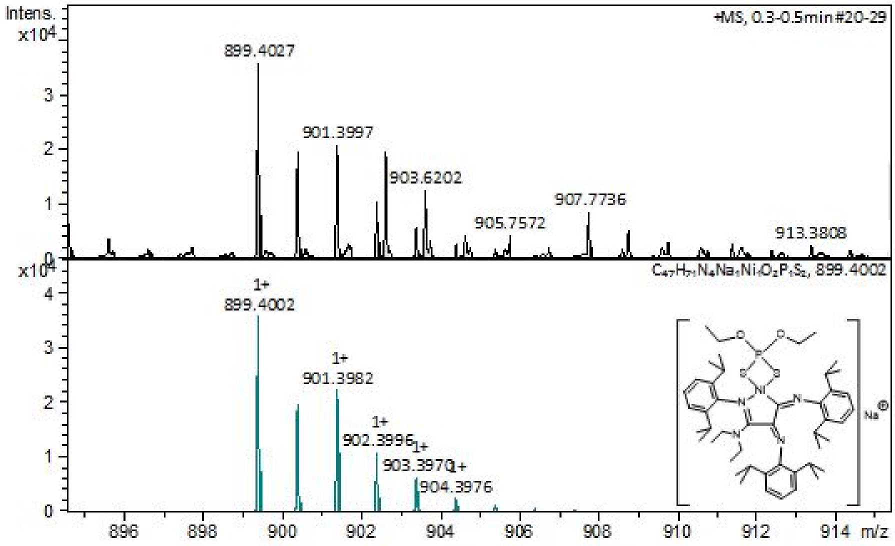

Meas. m/z	m/z	Err. [ppm]	m. sigma	Ion formula
899.4027	899.4002	2.8	19.6	$C_{47}H_{71}N_4NaNiO_2PS_2$

Figure S35. *HR-MS (ESI-TOF) of* ***3a***.

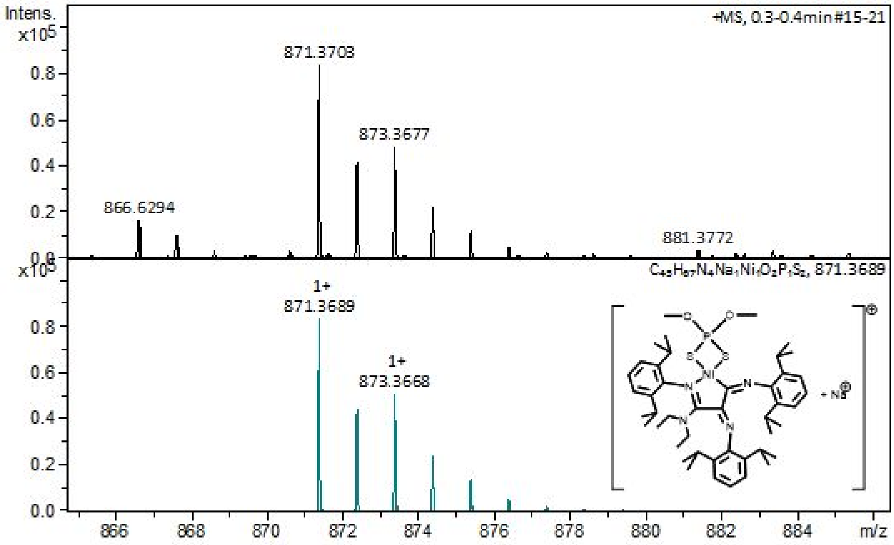

Meas. m/z	m/z	Err. [ppm]	m. sigma	Ion formula
871.3703	871.3689	-1.6	22.4	$C_{45}H_{67}N_4NiO_2PS_2$

Figure S36. *HR-MS (ESI-TOF) of* ***3b***.

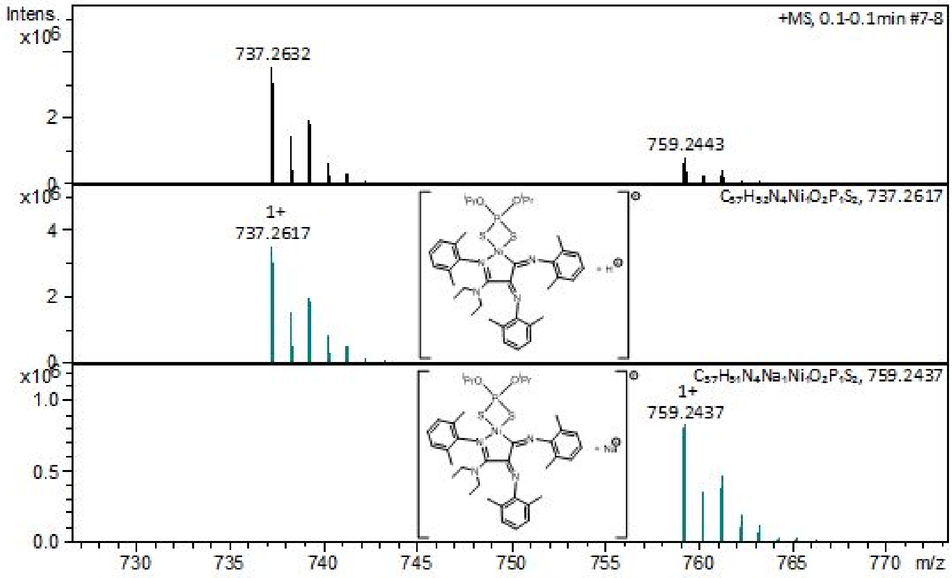

Meas. m/z	m/z	Err. [ppm]	m. sigma	Ion formula
737.2632	737.2617	-2	25.4	$C_{37}H_{52}N_4NiO_2PS_2$
759.2443	759.2437	-0.8	34.1	$C_{37}H_{52}N_4NaNiO_2PS_2$

Figure S37. *HR-MS (ESI-TOF) of* ***3d****.*

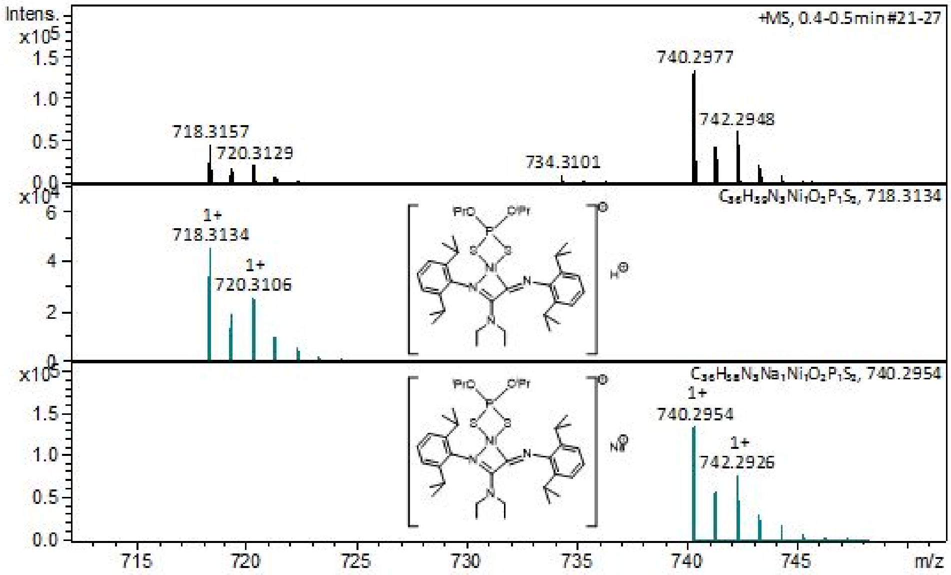

Meas. m/z	m/z	Err. [ppm]	m. sigma	Ion formula
718.3157	718.3134	3.1	45.1	$C_{36}H_{59}N_3NiO_2PS_2$
740.2977	740.2954	3.2	63.5	$C_{36}H_{58}N_3NaNiO_2PS_2$

Figure S38*. HR-MS (ESI-TOF) of* ***4c****.*

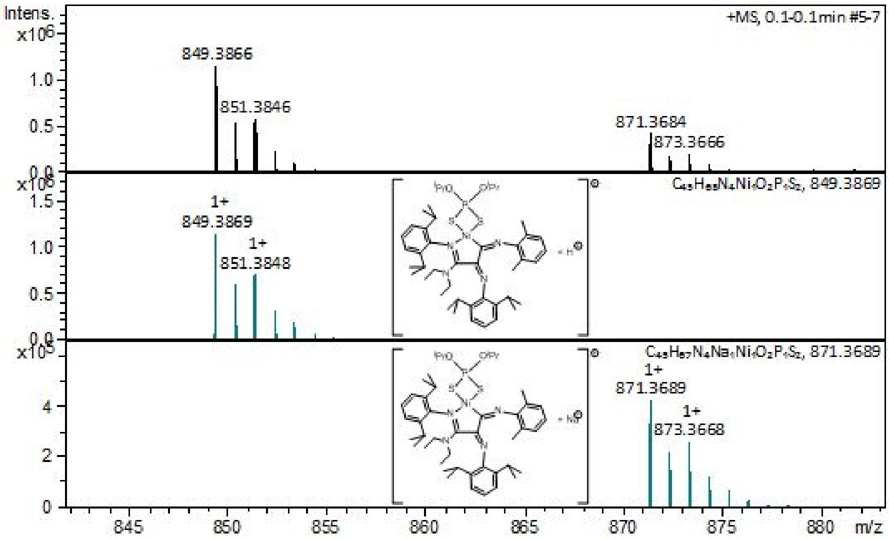

Meas. m/z	m/z	Err. [ppm]	m. sigma	Ion formula
849.3866	849.3869	0.4	54.9	$C_{45}H_{68}N_4NiO_2PS_2$
871.3684	871.3689	-0.5	61.30	$C_{45}H_{67}N_4NaNiO_2PS_2$

***Figure S39**. HR-MS (ESI-TOF) of **5**.*

X-Ray Crystallographic Studies.

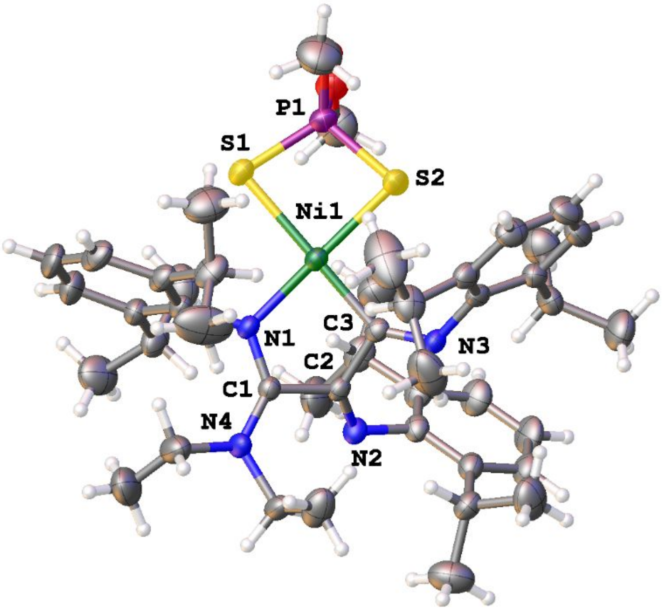

Figure S40. *X-Ray diffraction structure of* ***3b***. *Selected bond lengths (Å) and angles (deg): Ni(1)–S(1): 2.3449(10), Ni(1)–S(2): 2.2280(11), Ni(1)–N(1): 1.951(3), Ni(1)–C(3): 1.887(3), S(1)–Ni(1)–S(2): 86.31(4), N(1)–Ni(1)–C(3): 85.28(12).*

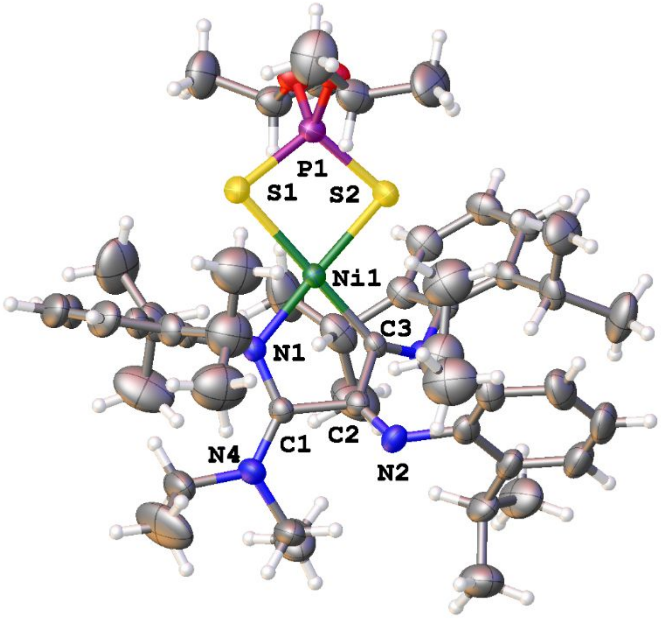

Figure 41. *X-Ray diffraction structure of* ***3c***. *Selected bond lengths (Å) and angles (deg): Ni(1)–S(1): 2.3339(12), Ni(1)–S(2): 2.2164(11), Ni(1)–N(1): 1.938(3), Ni(1)–C(3): 1.880(4), S(1)–Ni(1)–S(2): 86.57(4), N(1)–Ni(1)–C(3): 85.03(15).*

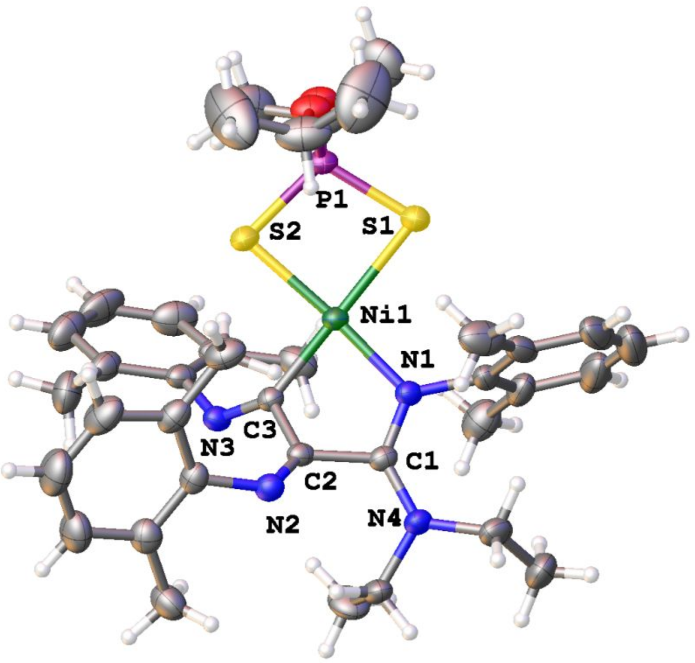

Figure S42. *X-Ray diffraction structure of **3d**. Selected bond lengths (Å) and angles (deg): Ni(1)–S(1): 2.3319(9), Ni(1)–S(2): 2.2179(9), Ni(1)–N(1): 1.939(2), Ni(1)–C(3): 1.883(3), S(1)–Ni(1)–S(2): 87.11(3), N(1)–Ni(1)–C(3): 84.56(11).*

Compounds 3b, 3c and 3d: The structures of complexes **3b**, **3c** and **3d** were fully elucidated using X-ray diffraction. For all the complexes, the open envelope structure is again observed in the nickelcyclopentene framework. The C(3) atom is out of the plane defined by Ni (1), C(1) and N(1) with the deviation from the plane being 0.271(8) Å, 0.343(9) Å and 0.522(6) Å for compounds **3b**, **3c** and **3d**, respectively. The bite angles of N(1)–C(3)–Ni(1) are 85.28(12)°, 85.03(15)° and 84.56(11)°, respectively. The torsion angle of S(2)–Ni(1)–C(1)–C(2) is 156.56(17) °, 151.94(18)° and 143.68(18)° for compounds **3b**, **3c** and **3d**, respectively. The Ni(1)–C(3) distances are below the sum of covalent radii[1] and are also similar to that of **3a** and other previously reported structures.[2-3]

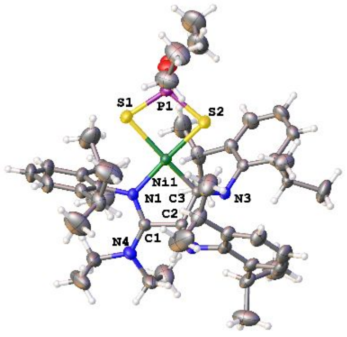

Compound 3a: $C_{47}H_{71}N_4NiO_2PS_2$ (*M* = 877.87 g/mol): triclinic, space group *P*-1 (no. 2), *a* = 11.4182(4) Å, *b* = 11.6495(4) Å, *c* = 19.6451(7) Å, *α* = 83.447(3)°, *β* = 75.896(3)°, *γ* = 76.939(3)°, *V* = 2463.96(16) Å^3, *Z* = 2, *T* = 293(2), K, μ(Mo Kα) = 0.550 mm^{-1}, Dcalc = 1.183 g/cm^3, 19404 reflections measured (5.024° ≤ 2Θ ≤ 59.562°), 11556 unique (R_{int} = 0.0231, R_{sigma} = 0.0504) which were used in all calculations. The final R1 was 0.0568 (*I* > 2σ(*I*)) and wR2 was 0.1549 (all data). CCDC: 2405809.

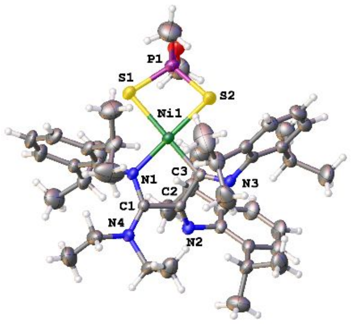

Compound 3b: $C_{45}H_{67}N_4NiO_2PS_2$ (*M* = 849.82 g/mol): monoclinic, space group *P*2$_1$/*n* (no. 14), *a* = 12.5609(5) Å, *b* = 18.9108(6) Å, *c* = 20.3865(7) Å, *β* = 92.215(3)°, *V* = 4838.9(3) Å^3, *Z* = 4, *T* = 293(2) K, μ(Mo Kα) = 0.558 mm^{-1}, Dcalc = 1.167 g/cm^3, 26376 reflections measured (5.394° ≤ 2Θ ≤ 59.326°), 11546 unique (R_{int} = 0.0499, R_{sigma} = 0.0858) which were used in all calculations. The final R_1 was 0.0624 (*I* > 2σ(*I*)) and wR_2 was 0.1798 (all data). CCDC: 2407041

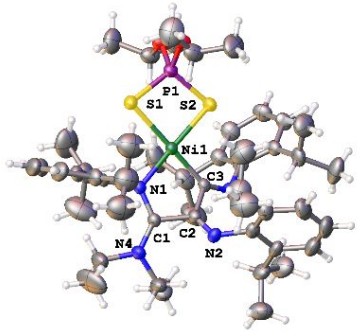

Compound 3c: $C_{49}H_{75}N_4NiO_2PS_2$ (*M* = 905.93 g/mol): monoclinic, space group *P*2$_1$/*n* (no. 14), *a* = 24.5205(6) Å, *b* =18.3058(4) Å, *c* = 24.9744(5) Å, *β* = 110.893(3)°, *V* = 10473.0(5) Å^3, *Z* = 8, *T* = 293(2), K, μ(Mo Kα) = 0.519 mm^{-1}, *Dcalc* = 1.149 g/cm^3, 115988 reflections measured (4.194° ≤ 2Θ ≤ 59.496°), 26255 unique (R_{int} = 0.0972, R_{sigma} = 0.1205) which were used in all calculations. The final R_1 was 0.0727 (*I* > 2σ(*I*)) and wR_2 was 0.2093 (all data). CCDC: 2407042

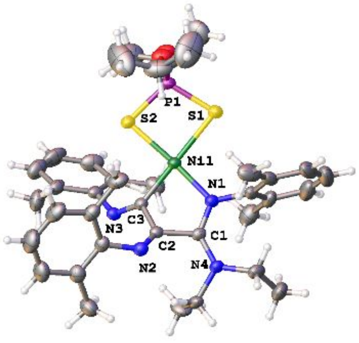

Compound 3d: $C_{37}H_{51}N_4NiO_2PS_2$ (M=737.61 g/mol): monoclinic, space group $P2_1/c$ (no. 14), a = 12.6902(5) Å, b = 10.9243(4) Å, c = 28.6121(13) Å, β = 97.480(4)°, V = 3932.8(3) Å^3, Z = 4, T = 293(2) K, μ(Mo Kα) = 0.676 mm^{-1}, $Dcalc$ = 1.246 g/cm^3, 19229 reflections measured (5.26° ≤ 2Θ ≤ 59.596°), 9374 unique (R_{int} = 0.0431, R_{sigma} = 0.0799) which were used in all calculations. The final R_1 was 0.0538 (I > 2σ(I)) and wR_2 was 0.1179 (all data). CCDC: 2405865.

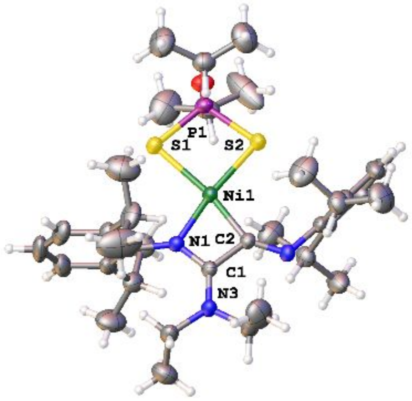

Compound 4c: $C_{36}H_{58}N_3NiO_2PS_2$ (M=718.65 g/mol): monoclinic, space group $C2/c$ (no. 15), a = 36.6479(8) Å, b = 10.2081(3) Å, c = 21.8721(6) Å, β = 96.049(2)°, V = 8136.9(4) Å^3, Z = 8, T = 293(2) K, μ(Mo Kα) = 0.651 mm^{-1}, $Dcalc$ = 1.173 g/cm^3, 88566 reflections measured (4.154° ≤ 2Θ ≤ 59.366°), 10757 unique (R_{int} = 0.0882, R_{sigma} = 0.0688) which were used in all calculations. The final R_1 was 0.0570 (I > 2σ(I)) and wR_2 was 0.1548 (all data). CCDC: 2405866.

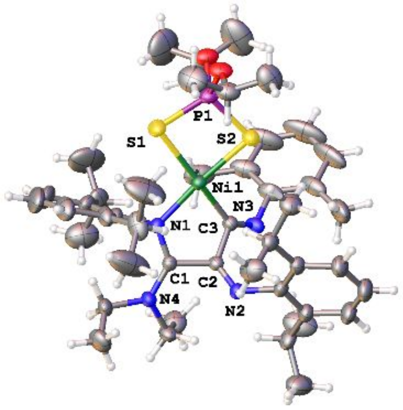

Compound 5: $C_{45}H_{67}N_4NiO_2PS_2$ (M=849.82 g/mol): monoclinic, space group $P2_1/n$ (no. 14), a = 11.2898(5) Å, b= 37.4683(17) Å, c = 11.4371(4) Å, β = 103.008(4)°, V = 4713.9(3) Å^3, Z = 4, T = 293(2) K, μ(Mo Kα) = 0.573 mm^{-1}, $Dcalc$ = 1.197 g/cm^3, 26487 reflections measured (4.252° ≤ 2Θ ≤ 59.212°), 11248 unique (R_{int} = 0.0417, R_{sigma} = 0.0691) which were used in all calculations. The final R_1 was 0.0680 (I > 2σ(I)) and wR_2 was 0.2014 (all data). CCDC: 2405872.

References

1. Cordero, B.; Gomez, V.; Platero-Prats, A. E.; Reves, M.; Echeverria, J.; Cremades, E.; Barragan, F.; Alvarez, S., Covalent radii revisited. *Dalton Trans.* **2008,** (21), 2832-8.
2. Noda, D.; Tanabiki, M.; Tsuchiya, K.; Sunada, Y.; Nagashima, H., Mono- and bimetallic ethylene polymerization catalysts having an azanickellacyclopentene skeleton. *Polyhedron* **2009,** *28* (18), 3935-3944.
3. Carmona, E.; Marín, J. M.; Palma, P.; Poveda, M. L., Multiple insertion reactions of CNBu' into nickel-alkyl bonds. *J. Organomet. Chem.* **1989,** *377* (1), 157-169.
4. David R. Lide, ed., CRC Handbook of Chemistry and Physics, Internet Version 2005, <http://www.hbcpnetbase.com>, CRC Press, Boca Raton, FL, **2005**.
